# Leveraging high-throughput analytics and automation to rapidly develop high-concentration mAb formulations: integrated excipient compatibility and viscosity screening

**DOI:** 10.1093/abt/tbae028

**Published:** 2024-10-12

**Authors:** Lun Xin, Lan Lan, Mourad Mellal, Nathan McChesney, Robert Vaughan, Claudia Berdugo, Yunsong Li, Jingtao Zhang

**Affiliations:** Product Development, Catalent Pharma Solutions, 3770 W. Jonathan Dr., Bloomington, IN 47404, United States; Product Development, Catalent Pharma Solutions, 3770 W. Jonathan Dr., Bloomington, IN 47404, United States; Product Development, Catalent Pharma Solutions, 14 School House Rd, Somerset, NJ 08873, United States; Product Development, Catalent Pharma Solutions, 3770 W. Jonathan Dr., Bloomington, IN 47404, United States; Product Development, Catalent Pharma Solutions, 3770 W. Jonathan Dr., Bloomington, IN 47404, United States; Product Development, Catalent Pharma Solutions, 3770 W. Jonathan Dr., Bloomington, IN 47404, United States; Product Development, Catalent Pharma Solutions, 3770 W. Jonathan Dr., Bloomington, IN 47404, United States; Product Development, Catalent Pharma Solutions, 14 School House Rd, Somerset, NJ 08873, United States

**Keywords:** high-concentration antibody formulation, excipient compatibility, automated buffer exchange and ultrafiltration, melting temperature, colloidal interaction, stability, viscosity, multiple linear regression

## Abstract

Background: Formulation screening is essential to experimentally balance stability and viscosity in high-concentration mAb formulations. We developed a high-throughput approach with automated sample preparation and analytical workflows to enable the integrated assessment of excipient compatibility and viscosity of mAb formulations. Methods: Ninety-six formulations of a trastuzumab biosimilar were screened by combining 8 types of excipient modifiers with 4 types of buffers across a pH range of 4.5 to 7.5. Key stability risks, including high molecular weight (HMW) aggregation and fragmentation, were thoroughly assessed along with viscosity at high concentrations. Additionally, several biophysical parameters were evaluated for their ability to predict stability or viscosity outcomes. Multiple linear regression was applied to fit the data and identify key factors. Results: The optimal pH range for the trastuzumab biosimilar was found to be 5.0 to 6.5, based on opposing pH dependencies for stability and viscosity. Buffer type had a minor effect on viscosity and fragmentation but played a significant role in influencing HMW aggregates, with Na-acetate and histidine-HCl being the best candidates. The impact of excipient modifiers on viscosity, HMW, and fragmentation depended on both pH and buffer type, showing strong interactions among factors. Arginine-HCl and lysine-HCl effectively lowered viscosity of the trastuzumab biosimilar at pH levels above 6.0, while glycine formulations were more effective at reducing viscosity below pH 6.0. Histidine-HCl, arginine-HCl, and lysine-HCl lowered the risk of HMW aggregation, whereas formulations containing Na-phosphate or NaCl showed higher HMW aggregation. Formulations with arginine-HCl, lysine-HCl, and NaCl demonstrated a rapid increase in fragmentation at pH levels below 5.0, while Na-aspartate formulations showed increased fragmentation at pH levels above 6.5. Conclusion: Hence, it is important to optimize the levels of each chosen excipient in the formulation study to balance their benefits against potential incompatibilities. This study serves as a foundation for identifying high-concentration antibody formulations using a high-throughput approach, where minimal materials are required, and optimized formulation design spaces can be quickly identified.

## Teaser

Statement of Significance: High-throughput screening has become an important approach for formulators to rapidly and robustly identify operational spaces for antibody therapeutics. This work describes a fully integrated high-throughput solution for high-concentration trastuzumab biosimilar formulation screening using readily available instruments. The effort can help to accelerate the adoption of high-throughput methodologies in mAb formulation development.

## Introduction

The development and subsequent commercialization of monoclonal antibody (mAb) have revolutionized treatments for oncology, immunology, infectious, and neurological diseases. The significant growth of this product class and related molecular constructs presents unique challenges and opportunities for pharmaceutical development. For example, while intravenous (IV) administration remains the most common method for delivering mAbs, subcutaneous (SC) injection has emerged as a more favorable route [1, 2]. IV delivery provides rapid and complete absorption of mAbs but requires dedicated infrastructure (e.g., hospitals or infusion centers) and trained personnel for administration. In contrast, SC injection allows for patient self-administration, enhances patient comfort and compliance, shortens administration time, and reduces healthcare costs [1]. Nevertheless, developing a product for SC delivery presents several key challenges: incomplete absorption of mAbs through the SC route, a potential increase in immunogenicity, and the need for high dose and/or high-concentration formulations [1–3]. Although there are sophisticated device approaches to increase the injection volume for SC delivery (e.g., via SC infusion), the current volume limit for a simple bolus syringe injection is approximately 2.0 mL [4]. This limitation often requires high-concentration (50-150 mg/mL) or ultra-high concentration (>150 mg/mL) mAb formulations to meet dosing targets [5, 6].

**Figure 1:**
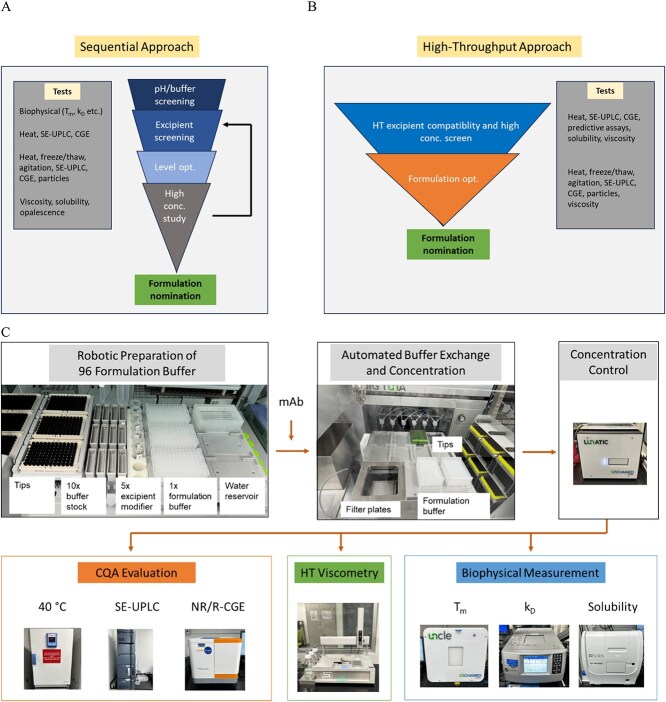
Typical workflow for mAb formulation development. A) Sequential approach that is traditionally used in the industry. B) High-throughput approach that can reduce time and allow the study of interactions among formulation parameters. C) Implemented automation workflow for high-throughput formulation screening (see methods for details).

The development of high concentration antibody formulation can be lengthy and resource intensive, as it’s a multi-attribute optimization problem with numerous input variables that can interact with each other (i.e. high dimensionality). All protein formulations must meet product stability requirements (e.g. biochemical and biophysical). The critical attributes for product stability are specific to each product but may include HMW aggregates, fragments, charge variants, oxidation products, and particles. Additionally, high-concentration formulations must address unique physicochemical challenges, such as high viscosity, high injection force, phase separation, and turbidity [2]. To address these challenges, pharmaceutical scientists typically screen combinations of solution components (e.g. pH, buffers, excipient modifiers, surfactants) to balance stability and physicochemical properties [5, 7]. Although rational design of protein formulations is desirable, practical development tends to be empirical due to factor interactions, uncertainties in excipient compatibility, and insufficient mechanistic understanding. Traditional formulation development for high-concentration products often relies on sequential screening due to constraints in material availability and analytical throughput (Fig. 1A) [7]. This sequential approach builds on a typical workflow for low-concentration formulations and adds a high-concentration study after determining the operational space of formulation stability. Since mAb stability testing is both time- and material-consuming, predictive biophysical parameters (e.g., T_m_, T_agg_, k_D_, B_22_, solubility) are often incorporated to assist formulation selection [8]. Nevertheless, the predictive effectiveness of these parameters for high-concentration product formulations is not fully validated. Furthermore, the number of formulations in each screening step is typically limited to fewer than 20 due to manual operations. Notably, the sequential screening approach is often used for SC formulation development of late-stage products, where stability insights have already been gained from early-stage IV formulation development. However, this approach can be highly iterative and time-consuming if the high-concentration properties of the formulation are found insufficient and require alternative excipients, leading to changes in stability profiles and additional excipient screening [5]. This approach is fundamentally suited for systems with additive properties and less effective for those with strong interactions.

High-throughput (HT) screening is a crucial approach in modern pharmaceutical development, as it reduces sample consumption, shortens the development timelines, and increases robustness of decision-making. HT screening for protein formulations typically relies on plate-based sample preparation, robotic liquid handling, and highly sensitive analytics to rapidly assess up to 96 conditions involving pH, buffer types, and stabilizing excipients [9–15]. Importantly, complex interactions among these factors can be elucidated at the end of experiments by applying statistical methods [11, 12]. Applying HT screening to high-concentration mAb development poses additional challenges, such as high material requirements, difficulties in preparing high-concentration samples, and the lack of integrated workflows. High-concentration samples in various formulation conditions are traditionally prepared using bench-scale ultrafiltration-diafiltration (UF/DF) devices, centrifugal ultrafiltration, dialysis, or direct spiking [11]. However, these methods are labor-intensive and not suited for HT implementation. Moreover, analytical tests for high- concentration behavior can consume large sample quantities (e.g. several hundred milligrams materials for a single viscosity measurement). Ying et al. developed a plate-based buffer exchange method to support formulation preparation for HT experiments [14], while Ren et al. recently reported a custom-built system to prepare and analyze high-concentration mAb samples for HT formulation studies [15]. Although these studies demonstrated significant efficiency gains with an HT setup, broader adoption has been limited by the custom-built nature of these systems and the resulting challenges in system integration.

We developed a fully integrated HT solution for high-concentration mAb formulation screening using readily available instruments and data solutions (Fig. 1C). The system consists of stations for robotic formulation matrix preparation, automated buffer exchange and sample concentration, HT measurement of biophysical parameters, low-volume HT viscosity measurement, and HT stability measurement. With this automation setup, we propose to reduce the formulation screening studies from four (Fig. 1A) to two (Fig. 1B), allowing up to 96 samples to be prepared and tested simultaneously in each study. These HT samples will contain various combinations of pH, buffers, and excipient modifiers, directly supporting the study of interactions. Reducing the number of studies and incorporating high-concentration assessments in the initial screening phase can minimize iterations, thereby shortening the development timeline. While the system allows for flexibility in implementation, we focus the first study on excipient compatibility and viscosity assessment, with a future study on formulation optimization. The initial screen aims to identify opportunities for viscosity reduction and gather sufficient stability data to eliminate potential liabilities. In contrast, the formulation optimization study will concentrate on a subset of screened conditions to further refine product stability at relevant concentrations and address additional needs for viscosity reduction.

Trastuzumab was initially developed as a lyophilized IV product for treating HER-2 positive breast cancer. Recently, an SC formulation of trastuzumab, marketed as Herceptin HYLECTA, was approved, but required the use of hyaluronidase to allow for larger dosing volumes. In this study, a trastuzumab biosimilar was used to test high-concentration formulation development workflow, with a focus on excipient compatibility and viscosity. We assessed the effectiveness of conformational and colloidal stability assays in predicting product stability as well as viscosity at high concentrations, as these assays are commonly utilized in formulation studies. Additionally, we evaluated several in-process measurements during filtration and determined their correlations with colloidal stability parameters and viscosity. To understand the impact of formulations on critical quality attributes (CQAs) and elucidate degradation mechanisms, we also compared orthogonal analytical methods and determined their effects on each CQA. This study lays the groundwork for identifying high concentration antibody formulations using an HT approach, where minimal materials are required, and optimized formulation design spaces can be rapidly identified.

## Results and discussion

### High-throughput formulation screening study design

High-throughput (HT) formulation development was reported previously with primary focus on biophysical parameters and protein stability under stressed or accelerated degradation conditions [9–12]. To support high-concentration formulation study, we tested the HT methodologies of preparing high concentration samples and measuring viscosity. Hence, current HT formulation screening includes biophysical assays, stability-indicating assays, and viscosity measurements (Fig. 1C). Stability-indicating assays will evaluate key product quality attributes including HMW aggregation and fragmentation using several orthogonal analytical tests. Biophysical assays are measurements for conformational and colloidal stability as well as protein solubility and selected process parameters during product concentration.

**Figure 2:**
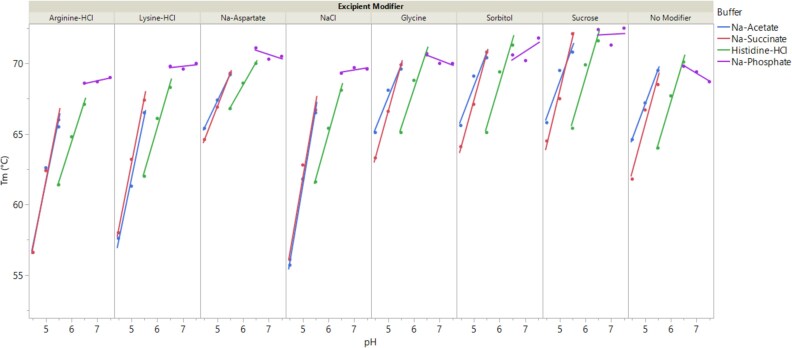
Impact of formulation conditions on the melting temperature (T_m_) of trastuzumab biosimilar. Data are shown as experimental values (solid symbols) overlaid with fitted lines to guide data reading. Each formulation consists of a 20 mM buffer at the target pH and an excipient modifier (see methods for details on formulation conditions). Control formulation with only buffer and no excipient modifier is labeled as No modifier. Formulations consisting of the same buffer are labeled using the same color (blue: Na-acetate; red: Na-succinate; green: Histidine-HCl; purple: Na-phosphate).

Instead of sequentially screening formulation factors such as pH, buffer, and excipient modifiers (Fig. 1A), this study aims to directly assess a formulation matrix comprising pH, buffers, and excipient modifiers for a more holistic evaluation of their impact on stability and viscosity (Fig. 1B). To achieve this, we designed a 96-well excipient plate that orthogonally pairs pH and buffer systems with excipient modifiers commonly used in protein formulations [16]. For example, seven modifiers, including tonicity agents, cryoprotectants, and viscosity reduction agents, were paired with 12 combinations of pH/buffer systems (Table S1 and Fig. 2). The levels of the excipients were selected at estimated isotonic levels for easier comparison. The pH/buffer systems included both monovalent and divalent buffers in the range of pH 4.5 to 7.5, as these frequently had the largest impact on product stability and viscosity. As is common practice, the pH of formulations was chosen to be close to the buffer’s pKa value to maximize buffer capacity. To directly compare buffer effects, several formulations shared the same pH but used different buffers. Additionally, modifiers from different excipient classes were selected to assess the effects of ionic, hydrogen bonding, hydrophobic, or π-π interactions. Control groups with no excipient modifier were also included to evaluate the positive or negative contributions of the excipient modifiers. Surfactants were excluded from this screen and will be addressed in future formulation optimization studies due to their effect on interfacial stress rather than viscosity. Product viscosities were assessed at high concentrations (125 mg/mL), while biophysical and stability testing were performed at reduced concentrations to be compatible with instrumentation and conserve materials. This HT screening platform enabled us to generate a comprehensive dataset for up to 96 formulation conditions using less than 2 grams of mAb within 6 weeks (Table S2). To facilitate data analysis, multiple linear regression analysis was employed to delineate the impact of formulation parameters on biophysical parameters, stability, and viscosity. Additionally, statistical analysis was used to determine the correlations between different biophysical measurements and their effectiveness in predicting the CQA, such as stability or viscosity.

### Conformational stability measurement

Conformational stability is a crucial design consideration in protein formulation development. Various tools can measure this stability, including differential scanning calorimetry (DSC), differential scanning fluorimetry (DSF), and isothermal chemical denaturation (ICD) [8, 13]. Among these, intrinsic tryptophan (Trp) DSF is particularly suited for HT screening, as it uses intrinsic fluorescence to measure unfolding. Intrinsic Trp DSF was used to assess the conformational stability of the trastuzumab biosimilar across 96 formulation conditions.

Figure 2 illustrates the dependence of T_m_ on formulation conditions, plotted as a function of pH and categorized by buffer types and excipient modifiers. The data show that T_m_ strongly depended on formulation pH. For instance, T_m_ increased with rising pH in formulations containing buffers like Na-acetate, Na-succinate, and histidine-HCl, regardless of the excipient modifiers used. Conversely, formulations with Na-phosphate exhibited relatively high T_m_ values with minimal variation across the pH range (6.5-7.5), indicating a plateau effect for the combination conditions of pH and Na-phosphate. Multiple linear regression was applied to fit the data and assess the statistical significance of these observations along with other formulation factors (Fig. S1). The model, including individual factors (pH, buffer, and excipient modifier) and selected two-way interactions (pH x buffer, pH x excipient modifier), achieved an R^2^ value of 0.97, indicating a strong fit. Scaled parameter estimates with significance were used to gauge the contribution of these factors (Table S3). Significant factors with the most positive impact on T_m_ (i.e., increasing T_m_) were pH, pH x Na-succinate, sucrose, and pH x NaCl, in descending order. Conversely, factors with the most negative impact on T_m_ were pH x Na-phosphate, histidine-HCl, arginine-HCl, NaCl, and lysine-HCl, in descending order. Examination of Fig. 2 reveals that samples with arginine-HCl, lysine-HCl, and NaCl had notably lower T_m_, while sucrose-containing samples had higher T_m_, consistent with their scaled estimate values. In addition, at pH 5.5, formulations with histidine-HCl had distinctly lower T_m_ compared to those with Na-acetate or Na-succinate. The increased T_m_ observed with sucrose can be attributed to improved protein folding due to preferential exclusion of excipients from protein surfaces [7]. The reduced T_m_ at lower pH or in the presence of histidine HCl, arginine-HCl, lysine-HCl, or NaCl aligns with findings for other mAbs and may be due to charge interactions from amino acids or counterions [12, 17–20]. The cross-interaction parameters in the model also indicate that some excipient modifiers or buffers significantly affect the pH response of T_m_. For example, formulations with Na-succinate and NaCl displayed a steeper pH slope (pH x Na-succinate, pH x NaCl).

### Colloidal stability measurements

Protein–protein interactions (PPI) are hypothesized to influence protein self-association and may correlate with solution viscosity and product stability [8, 21]. Colloidal stability parameters, such as B_22_, diffusion interaction parameter (k_D_), and relative solubility, are frequently used to rank formulations [22–26]. However, since B_22_ and k_D_ measurements are typically conducted at low protein concentrations, they may not reliably predict solution viscosity at high protein concentrations, due to the presence of multi-body interactions [24–26]. Despite these limitations, two common colloidal stability parameters, k_D_ and protein solubility, were measured across the 96 formulation conditions. Their effectiveness in predicting the stability and viscosity of the trastuzumab biosimilar was also evaluated.

**Figure 3:**
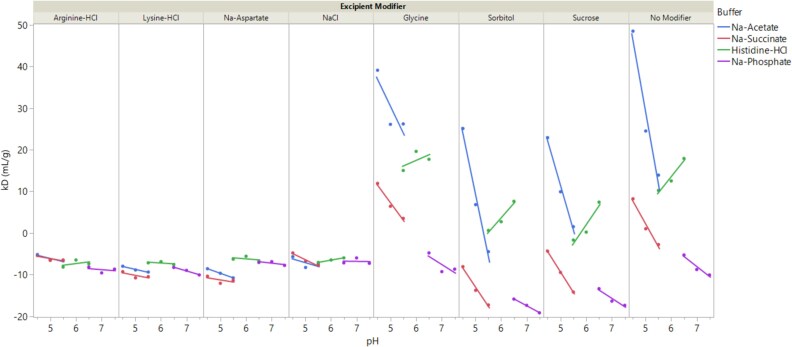
Impact of formulation conditions on diffusion interaction parameter (k_D_) of trastuzumab biosimilar. Data are shown as experimental values (solid symbols) overlaid with fitted lines to guide data reading. Each formulation consists of a 20 mM buffer at the target pH and an excipient modifier (see methods for details on formulation conditions). Control formulation with only buffer and no excipient modifier is labeled as No modifier. Formulations consisting of the same buffer are labeled using the same color (blue: Na-acetate; red: Na-succinate; green: Histidine-HCl; purple: Na-phosphate).

Figure 3 illustrates the dependence of k_D_ on formulation conditions, plotted as a function of pH and further classified by buffer type and excipient modifiers. As shown, the type of excipient modifiers played a significant role in determining k_D_ values. Two main classes of effects from excipients were observed. Excipients such as arginine-HCl, lysine-HCl, Na-aspartate, and NaCl resulted in formulations with negative k_D_ values, which remained relatively constant across pH and buffer types. These excipients contribute approximately 150 mM ionic strength to their respective formulations. In contrast, excipients such as glycine, sucrose, sorbitol, or the control with no modifier showed k_D_ values that ranged from positive to negative and were highly dependent on pH and buffer type. These excipients are either neutral or zwitterionic and do not contribute to ionic strength. Since PPI is heavily influenced by the electrostatic interactions between proteins, higher charges or stronger repulsive interactions are expected to lead to larger k_D_ values. Excipients with high ionic strength are likely to reduce electrostatic repulsion between protein molecules, resulting in lower k_D_. Further analysis of formulations with low ionic strength excipients showed that increasing pH in acidic buffer systems (such as Na-acetate, Na-succinate, and Na-phosphate) led to decreasing k_D_ values, while increasing pH in the basic buffer system (histidine-HCl) led to increasing k_D_ values. Since trastuzumab has an isoelectric point of ~ 9, it is expected to have a decreased charged state as pH increases in the range of pH 4.5-7.5. This trend is consistent with the expected changes in protein charge state as a function of pH in acidic buffers; however, it does not explain the findings in the histidine-HCl buffer system. Alternatively, the pH dependence of k_D_ in these buffer systems could be attributed to the ionic strength contributions from buffer ionization. For instance, as pH decreases, increased protonation of basic buffers like histidine-HCl will lead to higher ionic strength due to greater ionization of amines. In contrast, increased protonation of acidic buffers at lower pH results in lower ionic strength as the acid becomes neutral. Consistent with this, formulations with divalent buffers (Na-succinate and Na-phosphate) showed lower k_D_ values compared to those with monovalent buffers (Na-acetate and histidine-HCl), likely due to the higher ionic strength contributions from divalent ions at the same molar concentration. Taken together, the k_D_ of the trastuzumab biosimilar exhibited a strong dependence on ionic interactions, with excipient modifiers or buffers contributing to higher ionic strength predicted to have lower k_D_ values.

**Figure 4:**
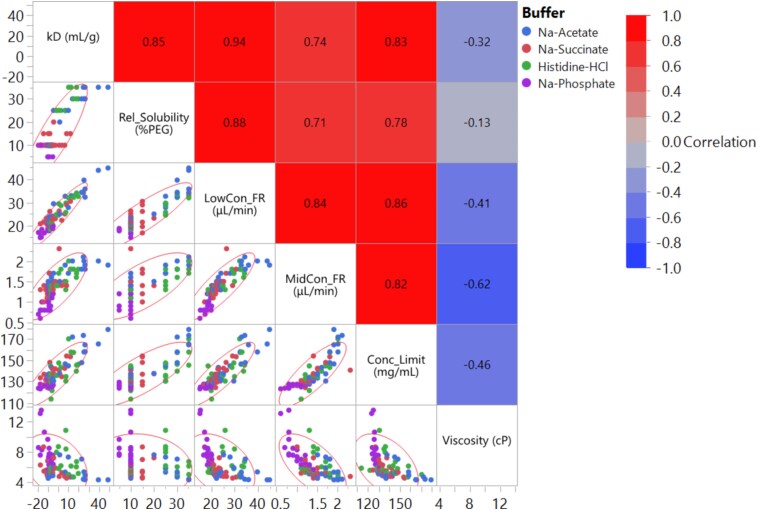
Correlation matrix for the diffusion interaction parameter (k_D_), relative solubility, buffer exchange in process measurements, and viscosity of trastuzumab biosimilar in different formulation conditions. Density ellipse with an alpha value of 0.95 was shown to guide the reading on the distribution of data. Formulations consisting of the same buffer are labeled using the same color in the correlation (blue: Na-acetate; red: Na-succinate; green: Histidine-HCl; purple: Na-phosphate). Heatmap shows the level of correlation between variables with their Pearson correlation coefficients shown inside.

We further utilized PEG-6000 induced protein phase separation to assess protein solubility [22, 23]. Figure S2A shows the dependence of protein solubility on formulation conditions, plotted as a function of pH and further classified by buffer type and excipients. Interestingly, similar effects from excipients were observed for protein solubility as for k_D_. For example, formulations containing excipients with high ionic strength—such as arginine-HCl, lysine-HCl, Na-aspartate, and NaCl—exhibited low protein solubility. In contrast, formulations with low ionic strength values (e.g., those containing low ionic strength buffers like Na-acetate or histidine-HCl, and excipients such as glycine, sucrose, sorbitol, or no modifier control) demonstrated high protein solubility. A strong positive correlation was observed between solubility and k_D_, with a correlation coefficient of 0.85 (Fig. 4).

It is important to note that due to the complexity of high-concentration formulation, assays performed at lower concentrations might not accurately predict the behavior of high-concentration protein formulations. As part of the automated preparation process for high concentration formulations, several in-process measurement parameters were obtained during buffer exchange and concentration. These parameters included buffer exchange filtration flow rate (FR) at different protein concentrations—specifically, the flow rate at 20 mg/mL, LowCon_FR (Fig. S2B) and 75 mg/mL, MidCon_FR (Fig. S2C) —and the maximum achievable concentration (Conc_Limit, Fig. S2D) (see methods for details). Given that the filtration process likely involves protein–protein interactions and product viscosity, these parameters could be valuable in predicting colloidal stability and product viscosity [23]. Indeed, a strong positive correlation was observed between the buffer exchange flow rates at 20 mg/mL (LowCon_FR) and 75 mg/mL (MidCon_FR), and colloidal stability indicators such as k_D_ and relative solubility (Fig. 4). LowCon_FR demonstrated stronger correlations to k_D_ (0.94) and relative solubility (0.88) than MidCon_FR did (0.74 and 0.71 respectively), reflecting the increased non-ideality at high concentrations. Interestingly, Conc_Limit showed strong correlations with k_D_ (0.83), relative solubility (0.78), LowCon-FR (0.86), and MidCon_FR (0.82), even though it was measured at the highest product concentration. Our study demonstrated that in-process data from the automated buffer exchange system may serve as indicators of colloidal stability. Since flow rate is an inherent output of the automated buffer exchange, using this data as a proxy for colloidal stability data could potentially eliminate the need for separate k_D_ or protein solubility experiments, thereby reducing the resources for HT testing. However, it should be noted that at this stage, the filtration flow rates are empirical measurements reflecting complex protein–protein interactions as well as solution viscosity. Further studies are required to validate and enhance these findings.

### High-throughput viscosity screening

Viscometers like the cone-plate rheometer remain the gold standard for measuring the dynamic viscosity of viscous solutions. However, traditional viscometers require large sample volumes, making them unsuitable for HT experimentation. To address this limitation, newer techniques such as capillary electrophoresis instrument [27] and microfluidic chip-based rheometer [28] have been developed. These methods support low sample consumption and are compatible with automation. In particular, microfluidic chip-based devices are advantageous because they can measure viscosity at different shear rates [28]. In this study, a chip-based device was utilized to analyze the viscosity of 96 formulations using minimal amount of material.

**Figure 5:**
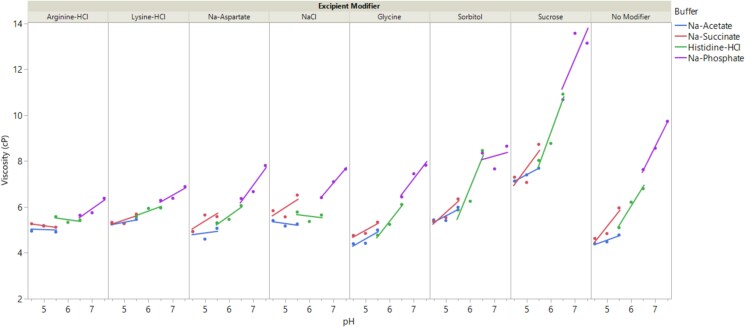
Impact of formulation conditions on the viscosity of the trastuzumab biosimilar at 125 mg/mL. Data are shown as experimental values (solid symbols) overlaid with fitted lines to guide data reading. Each formulation consists of a 20 mM buffer at the target pH and an excipient modifier (see methods for details on formulation conditions). Control formulation with only buffer and no excipient modifier is labeled as No modifier. Formulations consisting of the same buffer are labeled using the same color (blue: Na-acetate; red: Na-succinate; green: Histidine-HCl; purple: Na-phosphate).

Figure 5 illustrates the dependence of viscosity on formulation conditions, plotted as a function of pH and further classified based on buffer types and excipients. The data reveals that viscosity consistently increased with pH across all excipients. In addition, formulations containing sucrose exhibited higher overall viscosity values and steeper pH-dependent viscosity slopes compared to other formulations. Conversely, formulations with excipients like arginine-HCl and lysine-HCl displayed lower overall viscosity and gentler pH-dependent viscosity slopes when the pH is above 6.0. Formulations containing glycine showed lower viscosity when the pH is below 6.0. Multiple linear regression was performed to fit the data, considering individual factors (pH, buffer, and excipient modifier) along with a selected two-way interaction (pH x excipient modifier) (Fig. S3). The model achieved an R^2^ value of 0.95, indicating a strong fit. Scaled parameter estimates (Table S4) revealed that the factors with the most positive effects on viscosity were sucrose, pH x sucrose, pH, and pH x no modifier, in descending order. The factors with the most negative effects were pH x arginine-HCl, arginine-HCl, pH x lysine-HCl, glycine, pH x NaCl, Na-aspartate, lysine-HCl, also in descending order. Hence, the analysis supported the empirical observations made above on pH, sucrose, arginine-HCl, lysine-HCl, and glycine. The findings here align with previous reports that sucrose can increase the viscosity of mAb formulations at isotonic levels [29], though the mechanism by which sucrose affects the pH-dependent viscosity slope (compared to those formulations with no excipient modifier) remains unclear. The current observations regarding viscosity reduction by arginine-HCl, lysine-HCL, glycine, Na-aspartate are consistent with earlier studies demonstrating that certain amino acids and their salts can decrease the viscosity of high-concentration protein formulations [5, 18, 19, 30]. The reduction in the pH-dependent viscosity slope by these amino acids, particularly arginine-HCl and lysine-HCl (i.e. pH x arginine-HCl, pH x lysine-HCl), suggests the presence of charge–charge interactions. Interestingly, the buffer type appears to have a relatively minor influence compared to other factors like pH and the type of excipient modifier. A simpler model excluding buffer type (considering only factors of pH, excipient, and pH x excipient) yielded an R^2^ value of 0.93 (data not shown), further confirming that the viscosity of the trastuzumab biosimilar formulations is primarily governed by pH, excipient modifiers, and their interactions.

The correlation analysis between viscosity and colloidal stability parameters (Fig. 4) revealed a weak to moderate negative correlation (|r| < 0.7) across different parameters, including k_D_, relative solubility, LowCon_FR, MidCon_FR, and Conc_Limit. Among these, the buffer exchange flow rate at 75 mg/mL (MidCon_FR) showed the strongest correlation with viscosity at 125 mg/mL, with an r value of −0.62. The previously reported lack of correlation between viscosity and k_D_ [24–26] is primarily attributed to the multi-body interactions at high concentrations, which are not captured by low-concentration measurements by k_D_. The stronger correlation between MidCon_FR and viscosity compared to other parameters likely reflects the influence of viscosity on the filtration flow rate at higher concentrations. However, the moderate correlation coefficient (−0.62) suggests that the filtration process is affected by other factors beyond viscosity, such as membrane polarization. Overall, these findings emphasize the complexity involved in predicting the viscosity of high-concentration protein formulations. They also highlight the importance of directly measuring viscosity in these formulations, rather than relying solely on colloidal stability parameters measured at lower concentrations.

### Assessment of aggregation by orthogonal techniques

**Figure 6:**
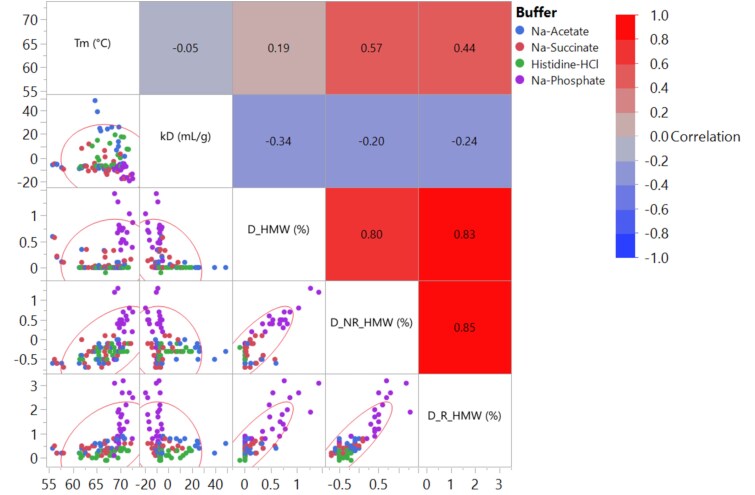
Correlation matrix for the melting temperature (T_m_), diffusion interaction parameter (k_D_), net increase in HMW as measured by SE-UPLC (D_HMW), net increase in HMW as measured by NR-CGE (D_NR_HMW), and net increase in HMW as measured by R-CGE (D_R_HMW) of the trastuzumab biosimilar in different formulation conditions. For HMW measurement, 5 mg/mL trastuzumab biosimilar formulations were stressed at 40 °C for 2 weeks and subsequently subjected to analytical testing to determine the relative change in HMW value. Density ellipse with an alpha value of 0.95 was shown to guide the reading on the distribution of data. Formulations consisting of the same buffer are labeled using the same color in the correlation (blue: Na-acetate; red: Na-succinate; green: Histidine-HCl; purple: Na-phosphate). Heatmap shows the level of correlation between variables with their Pearson correlation coefficients shown inside.

**Figure 7:**
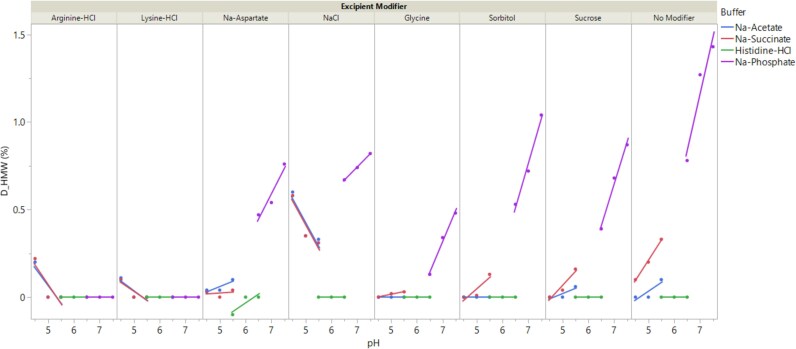
Impact of formulation conditions on the HMW aggregation of trastuzumab biosimilar. Trastuzumab biosimilar formulations at 5 mg/mL were stressed at 40 °C for 2 weeks and subsequently subjected to SE-UPLC to determine the net increase in HMW value. Data are shown as experimental values (solid symbols) overlaid with fitted lines to guide data reading. Each formulation consists of a 20 mM buffer at the target pH and an excipient modifier (see methods for details on formulation conditions). Control formulation with only buffer and no excipient modifier is labeled as No modifier. Formulations consisting of the same buffer are labeled using the same color (blue: Na-acetate; red: Na-succinate; green: Histidine-HCl; purple: Na-phosphate).

High molecular weight (HMW) aggregation is a CQA for protein products, and must be minimized under typical process, storage, and handling conditions. Aggregation of mAbs can be influenced by general protein aggregation mechanisms and specific excipient-protein interactions [18, 20, 31–36]. Thus, formulation development that screens combinations of pH, buffers, and excipient modifiers is essential to mitigate this risk. Although real-time stability data ultimately define product shelf life, accelerated or stress conditions (e.g., storage at 25 or 40 °C for a few weeks) are often employed to quickly screen formulations. For HT screening, we assessed the risk of HMW by incubating formulations at 40 °C for 2 weeks and then measuring the relative change in HMW value. Several methods exist to assess the HMW content of proteins, including size exclusion chromatography (SEC), analytical ultracentrifugation (AUC), and capillary gel electrophoresis (CGE). These methods are orthogonal in their analytical principles, and therefore, may not always report the same HMW value. For example, SEC generally characterizes samples in their native state and separates them by hydrodynamic volume on a column. It can detect both non-covalent and covalent aggregates. In contrast, CGE analyzes the samples under denatured conditions, separating by molar mass within a gel. It is less likely to detect non-covalent aggregates. Furthermore, CGE can be run under non-reduced (NR) or reduced (R) conditions to evaluate aggregates or fragments before and after cleavage of disulfide bonds. Given the need for HT-friendly methods, SE-UPLC, NR-CGE, and R-CGE were chosen to analyze the HMW aggregates across the 96 formulations. The correlation among different HMW measurement techniques (D_HMW, D_NR_HMW, D_R_HMW) was first assessed (Fig. 6). A strong correlation was observed among all three methods for the trastuzumab biosimilar formulations (|r| ≥ 0.80). This indicated that a large fraction of the HMW must be covalently associated. Additionally, the results show that the HMW risk of the trastuzumab biosimilar could be adequately described by a single technique. Given that SE-UPLC can also detect non-covalent aggregates, its results (i.e., D_HMW) were chosen as the primary data to evaluate the impact of formulation factors on HMW (Fig. 7). The HMW results from CGE measurements (D_NR_HMW, D_R_HMW) were provided in the supplementary materials (Fig. S4).

Figure 7 illustrates the dependence of HMW aggregates (D_HMW) on formulation conditions, plotted as a function of pH and further classified by buffer types and excipient modifiers. The results underscore the significant influence of both buffer and excipient types on HMW aggregation. Formulations based on histidine-HCl showed virtually no net increase in HMW after stress. This stabilization effect cannot be attributed solely to the pH range (pH 5.5-6.5) since formulations in other buffers, such as Na-phosphate, Na-acetate, and Na-succinate, at overlapping pH (pH 6.5 or 5.5) displayed much higher HMW values. The stabilizing effect of histidine-HCl buffer on the trastuzumab biosimilar is consistent with prior studies suggesting that histidine-HCl can stabilize other IgG products, potentially by blocking the hydrophobic patches on protein surfaces [32, 33, 35]. In contrast, Na-phosphate buffer showed a destabilizing effect, with most formulations exhibiting distinctly higher HMW values compared to those in other buffers. Additionally, the Na-phosphate formulations showed positive pH-HMW slopes, with both the HMW values and the pH-HMW slopes highly dependent on the excipient modifiers, indicating strong interactions between pH and excipients. Among them, formulations without excipients (no modifier group) had the largest HMW values and the most positive pH-HMW slopes, while those with arginine-HCl and lysine-HCl had no net increase in HMW. The underlying mechanism for the destabilizing effect by Na-phosphate is not entirely clear but could be mediated through the binding of divalent phosphate ions to charged residues on protein surfaces and the resulting ability to bridge two mAbs for aggregation [36]. The stabilizing effect by arginine-HCl and lysine-HCl cannot be explained by a general electrostatic screening mechanism, as other excipients with similar ionic strength (e.g., Na-aspartate and NaCl) resulted in much higher HMW values and pH-HMW slopes. In Na-acetate and Na-succinate buffers, formulations with NaCl had significantly higher HMW values compared to those with other excipients, suggesting a potential destabilization effect. Arginine-HCl and lysine-HCl might stabilize the trastuzumab biosimilar by specifically blocking the interaction of divalent phosphate ions with positively charged residues on the protein surface [20, 34, 36]. Recent studies have shown that counterions of arginine played a crucial role in inhibiting mAb aggregation [20]. Future studies need to be conducted to test whether other amine-based excipients or their counterions can be used to stabilize against HMW formation. Certain combinations of Na-acetate with glycine or sorbitol showed virtually no net increase in HMW after stress, whereas Na-succinate buffer with either glycine or sorbitol at similar pH ranges resulted in higher HMW values. This suggests a possible synergistic stabilizing effect when using Na-acetate in combination with glycine or sorbitol. Together, the data suggest a strong interaction between excipient and buffer in determining the aggregation of the trastuzumab biosimilar. A screening study as shown here is highly recommended to understand how excipient modifiers or individual buffer stabilize or destabilize mAb. Current analysis of data is mostly qualitative and focused on large differences where significance can be easily determined. A multiple liner regression model, incorporating individual factors and all two-way interactions, was used to fit the HMW data. The model achieved an R^2^ value of 0.98 and confirmed the importance of two-way interactions as the most important factors (data not shown). However, since most of the variability in the data were observed in Na-phosphate formulations (with no increase in HMW from histidine-HCl formulations), the model predominantly centered around Na-phosphate related factors, offering limited insights for other buffers or excipient modifiers.

Finally, correlation analysis between the HMW value and two key biophysical parameters—the melting temperature (T_m_) and diffusion interaction parameters (k_D_)—was performed for the trastuzumab biosimilar formulations, as illustrated in Fig. 6. Both parameters are crucial in understanding and predicting protein aggregation [37]. For instance, lower conformational stability may lead to increased protein unfolding, while lower colloidal stability can accelerate the kinetics of protein aggregation. However, correlation analysis revealed that T_m_ positively correlated with HMW, whereas k_D_ negatively correlated with HMW with a weak to moderate correlation coefficient (r = 0.34). These findings suggest that the aggregation behavior of mAbs in complex formulation matrixes cannot be fully described by such simple biophysical parameters alone. Although k_D_ might be a valuable tool to guide formulation design, it should not replace stability testing in formulation development.

### Assessment of fragmentation by orthogonal methods

**Figure 8:**
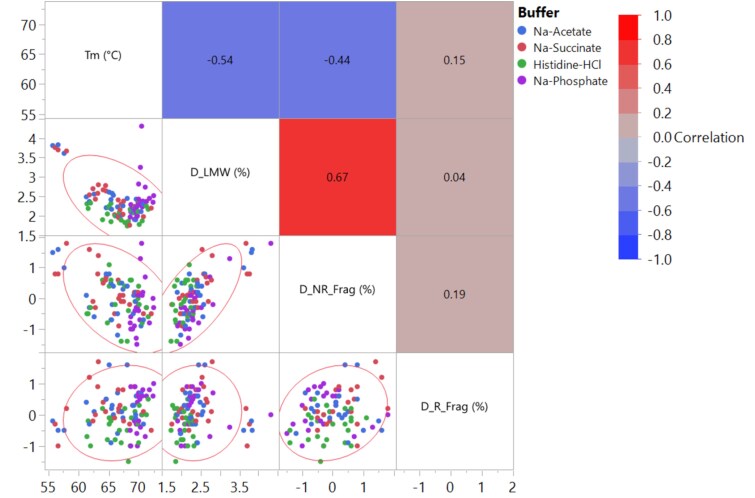
Correlation matrix for the melting temperature (T_m_), net increase in LMW as measured by SE-UPLC (D_LMW), net increase in fragmentation as measured by NR-CGE (D_NR_Frag), and net increase in fragmentation as measured by R-CGE (D_R_Frag) of trastuzumab biosimilar in different formulation conditions. For fragmentation measurement, trastuzumab biosimilar formulations at 5 mg/mL were stressed at 40 °C for 2 weeks and subsequently subjected to analytical testing to determine the relative change in fragmentation value. Density ellipse with an alpha value of 0.95 was shown to guide the reading on the distribution of data. Formulations consisting of the same buffer are labeled using the same color in the correlation (blue: Na-acetate; red: Na-succinate; green: Histidine-HCl; purple: Na-phosphate). Heatmap indicates the level of correlation between variables with their Pearson correlation coefficients shown inside.

Fragmentation is a CQA for IgG based protein products and is closely controlled during both manufacturing and storage. During drug substance manufacturing, IgG fragments primarily involve partially assembled heavy and light chains of mAbs. In contrast, stability testing often reveals IgG fragmentation due to the breakage in peptide or disulfide bonds, particularly in the hinge region due to chain mobility and exposure to the aqueous environment [38]. SEC and CGE are commonly used techniques to assess IgG fragmentation, providing complementary information. The late elution region of SEC (LMW) can be useful for characterizing hinge related fragments, though SEC may suffer from limited resolution and difficulty in resolving aggregated fragments. Non-reduced and reduced CGE separate based on molar mass and are ideally suited to determine fragments degraded from a parent molecule. Among them, non-reduced CGE is better suited to characterize the formation of hinge related fragments as reducing conditions can cause Fab fragments to co-migrate with light chain [39]. In this study, fragmentation of 96 formulations of the trastuzumab biosimilar were analyzed using SE-UPLC, NR-CGE, and R-CGE before and after incubation at 40 °C for 2 weeks. The correlations among the relative changes in fragmentation measured by these techniques (D_LMW, D_NR_Frag, D_R_Frag) were first assessed (Fig. 8). No significant correlations between D_R_Frag and the other two fragmentation measurements (D_LMW and D_NR_Frag, |r| < 0.2 for both) were observed, which may be attributed to artifacts induced by the reducing conditions (as discussed above) or lower signal-to-noise ratios for this technique. Indeed, D_R_Frag values were generally very small (<1%) and often negative for many formulations (Fig. S5). A moderate to strong correlation was found between D_LMW and D_NR_Frag (r = 0.67), indicating that both techniques can effectively track the formation of fragments. Nevertheless, the limited correlation also suggests that D_LMW and D_NR_Frag are orthogonal measurements, capturing different aspects of fragmentation process. Therefore, both D_LMW and D_NR_Frag were used to analyze the impact of formulation factors on fragmentation.

**Figure 9:**
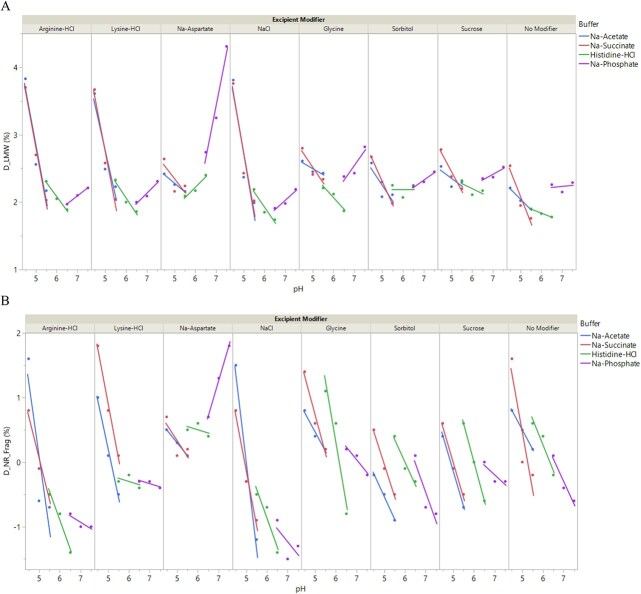
Impact of formulation conditions on the fragmentation of trastuzumab biosimilar. Trastuzumab formulations at 5 mg/mL were stressed at 40 °C for 2 weeks and subsequently subjected to analytical tests to determine the net increase in fragmentation value (panel a: Net increase in fragmentation as determined by SE-UPLC (D_LMW); panel B: Net increase in fragmentation as determined by NR-CGE (D_NR_Frag). Data are shown as experimental values (solid symbols) overlaid with fitted lines to guide data reading. Each formulation consists of a 20 mM buffer at the target pH and an excipient modifier (see methods for details on formulation conditions). Control formulation with only buffer and no excipient modifier is labeled as No modifier. Formulations consisting of the same buffer are labeled using the same color (blue: Na-acetate; red: Na-succinate; green: Histidine-HCl; purple: Na-phosphate).

Figure 9A illustrates the dependence of the net increase in SE-LMW (D_LMW) on formulation conditions, plotted as a function of pH and further classified by buffer type and excipient modifiers. The pH-LMW curve displayed a U-shaped trend with a minimum in the pH range of 5.5 to 6.5 for each excipient. The trastuzumab biosimilar formulations in Na-acetate and Na-succinate buffers exhibited more D_LMW fragments as the solution pH decreased below 5.5, resulting in negative pH-LMW slopes within this range. Among them, formulations containing arginine-HCl, lysine-HCl, and NaCl had markedly larger negative pH-LMW slopes compared to those with other excipients in the same buffer systems. Interestingly, all Na-phosphate buffer systems had low LMW values but showed positive pH-LMW slopes, except for formulations containing Na-aspartate, which demonstrated a larger positive slope. Formulations containing histidine-HCl overall exhibited lower LMW values, and less pH-dependent variation compared to those in other pH ranges. Multiple linear regression was used to fit the data and assess the statistical significance of these observations, as well as other formulation factors (Fig. S6). Several models were tested by selecting different cross interactions and reducing them to include only significant terms. The final model incorporated two individual factors (pH, excipient), a two-way interaction (pH x excipient), and a quadratic term for pH (pH x pH). The quadratic term accounted for the U-shaped pH response. This simple model achieved an R^2^ value of 0.86, indicating it sufficiently explained the variance in the data. Adding buffer or its cross interactions as factors only improved the R^2^ to 0.88, and their p-values were either non-significant or close to non-significant (p > 0.013). Therefore, buffer and its cross interactions were excluded from the final model. Scaled parameter estimates with significance were used to determine the contribution of various factors in the model (Table S5). Significant factors with the most positive effects on D_LMW were pH x Na-aspartate, pH x pH, and Na-aspartate, in descending order. Those with the most negative effects were pH x NaCl, pH x arginine-HCl, pH x lysine-HCl, pH, and no modifier, in descending order. These significant factors aligned with the above observations on pH and specific excipients (arginine-HCl, lysine-HCl, NaCl, Na-aspartate). The ability of such a simple model to describe the behavior of D_LWM suggests that fragmentation of the trastuzumab biosimilar is primarily influenced by pH and can be further modulated by a few excipients, such as arginine-HCl, lysine-HCl, NaCl, Na-aspartate. These findings are consistent with the fragmentation mechanisms known for specific IgG subtypes [38]. For instance, IgG1 kappa antibodies such as trastuzumab are prone to forming more hinge domain fragments at lower pH (<5) due to increased degradation of the Asp_221_-Lys_222_ bond in the upper hinge region. Similarly, at higher pH (>7), increased cleavage of the Ser_219_-Cys_220_ bond can occur via β-elimination, leading to the loss of light chains. Consequently, the optimal pH range to minimize fragmentation for IgG1 is typically between 5 and 7.

The impact of formulation factors on fragmentation was further evaluated using NR-CGE. Figure 9B shows the dependence of the net increase in NR_Frag (D_NR_Frag) on formulation conditions, plotted as a function of pH and further classified by buffer type and excipient modifiers. An overall trend similar to that of D_LMW was observed for formulations in Na-acetate and Na-succinate. In these formulations, increasing pH resulted in a reduction in D_NR_Frag levels. The pH-D_NR_Frag slopes varied with different excipients, with the most negative slopes observed for arginine-HCl, lysine-HCl, and NaCl. For Na-phosphate formulations, except for those containing Na-aspartate, they generally showed minimal variation in D_NR_Frag. Na-phosphate formulations containing Na-aspartate exhibited a large positive slope, similar to the patterns observed with D_LMW. In contrast, histidine-HCl formulations displayed greater pH-dependency compared to D_LMW. In particular, formulations containing glycine, sorbitol, or sucrose showed steep negative pH-D_NR_Frag slopes. The comparison between D_LMW and D_NR_Frag demonstrates that these two techniques provide complementary information and should be used together to assess the fragmentation risk of mAb. Lastly, correlations between melting temperature (T_m_) and the extent of fragmentation observed by D_LMW and D_NR_Frag were analyzed (Fig. 8). Lower conformational stability can lead to increased exposure of domains susceptible to fragmentation. Indeed, negative correlations were observed between T_m_ and fragmentation, although the correlation was moderate (0.3 ≤ |r| < 0.7). Nevertheless, this correlation helps to explain the negative impact of arginine-HCl, lysine-HCl, or NaCl on fragmentation at lower pH (Table S5), as formulations containing these excipients also exhibited lower melting temperatures (T_m_) compared to those with other excipients (Fig. 2).

## Conclusion

High-concentration antibody formulations are of significant interest to pharmaceutical developers, particularly for enabling the SC delivery of biologics. Comprehensive excipient screening is crucial to address the challenge of balancing stability and viscosity in these formulations. To this end, we developed a HT approach with automated sample preparation and analytical workflows, allowing for the integrated assessment of excipient compatibility and viscosity in mAb formulations. Using this approach, we explored 96 formulations of a trastuzumab biosimilar by combining 8 types of excipient modifiers with 4 buffers across a pH range of 4.5 to 7.5, maximizing the interactions between pH, buffer, and excipient modifier. Key stability risks, such as HMW aggregation and fragmentation, were thoroughly evaluated using multiple orthogonal techniques, while viscosity was assessed at relevant product concentrations. In addition, we assessed several biophysical parameters, including melting temperature (T_m_), diffusion interaction parameter (k_D_), protein solubility, filtration flow rates, to evaluate their predictive power for stability or viscosity outcomes. Multiple linear regression was employed to fit the data and identify key factors. This screening approach proved to be efficient, requiring less time and material than traditional sequential screening methods (Table S2). More importantly, it uncovered critical interactions and unique opportunities that might otherwise have been overlooked, enhancing the overall formulation development process.

Overall, pH, buffer type, excipient modifier, and their interactions played crucial roles in determining the CQAs of the trastuzumab biosimilar. pH was a central factor, influencing viscosity, HMW, and fragmentation, though in opposite directions: viscosity and HMW increased at higher pH, while fragmentation was more pronounced at lower pH. Therefore, the optimal formulation pH for the trastuzumab biosimilar likely falls within the middle of the screened range (pH 5.0-6.5). In contrast to pH, buffer type had a minor effect on viscosity and fragmentation but significantly influenced HMW aggregation. Among the four buffers tested, monovalent buffers like Na-acetate and histidine-HCl showed the least liabilities, with histidine-HCl emerging as particularly favorable due to its strong buffering capability in the desirable pH range (pK_a_ ~ 6) and its observed benefit in stabilizing against HMW aggregation. These findings align with the use of histidine-HCl in commercial trastuzumab formulations and its widespread application in other commercial mAb formulations.

Our study highlighted the nuanced balance required in formulation development by revealing both benefits and potential liabilities associated with various buffers and excipient modifiers. The pH-dependent trends in viscosity, HMW, and fragmentation were significantly influenced by the buffers and excipient modifiers. For instance, arginine-HCl and lysine-HCl effectively lowered viscosity of the trastuzumab biosimilar at pH levels above 6.0, while glycine formulations were more effective at reducing viscosity below pH 6.0. Arginine-HCl and lysine-HCl achieved this by moderating the pH-viscosity slope. Additionally, histidine-HCl, arginine-HCl, and lysine-HCl were found to mitigate the risk in HMW aggregation, thereby reducing its pH-dependency. In contrast, formulations containing Na-phosphate or NaCl exhibited higher levels of HMW aggregates, with responses heavily dependent on pH and excipients used. Furthermore, formulations with arginine-HCl, lysine-HCl, and NaCl showed a rapid increase in fragmentation at pH levels below 5.0, while Na-aspartate formulations demonstrated increased fragmentation above pH 6.5. These results underscore the complex interplay among formulation factors, emphasizing the need to carefully optimize the concentration of each chosen excipient to balance their benefits against potential incompatibilities. The precise choice and level of excipients must be tailored to the specific product attributes and specification requirements to achieve the desired stability and performance outcomes.

The interplay and complexities identified in this screening approach would be challenging to uncover through conventional sequential screening methods. In a sequential screen, where a pH/buffer screen is conducted first and followed by an excipient screen with a fixed pH/buffer, the liabilities and operational limit of the excipients within specific pH/buffer regions might not be immediately apparent. Recognizing these operational boundaries and liabilities early on can help to streamline the selection of pH/buffer combinations, leading to more robust formulations. This orthogonal matrix screen also offers the advantage of discovering formulations that might be overlooked in sequential screenings. For example, the formulation containing Na-acetate and glycine at a target pH 5.5 performed comparably to other histidine-HCl based formulations, highlighting the value of this approach in identifying alternative viable options. While design of experiment (DOE) methodologies can be employed to reduce the number of samples in high-throughput screenings, the time and resource savings may not be substantial. Due to the common practice of setting buffer pH near its pKa, pH and buffer type cannot be independently varied, necessitating a constrained DOE design where pH and buffer are treated as covariates. Such designs typically require larger sample sizes; for instance, a custom DOE design with covariates of pH and buffer in this study would require a minimum of 58 samples. Given the ease of automation to handle a 96-well orthogonal matrix design, the resource savings offered by a custom DOE design are limited, making the orthogonal matrix approach a more efficient option in this context.

Finally, biophysical parameters showed limited correlations with stability parameters or viscosity, although moderate correlations were observed between certain pairs, such as MidCon_FR and viscosity, k_D_ and HMW, and T_m_ and fragmentation. Therefore, it is recommended that while these biophysical parameters can be useful for guiding rational formulation design for excipient studies, they should not replace comprehensive stability testing. This preformulation screening approach has been instrumental in uncovering the complex interactions among pH, buffers, and excipient modifiers, as well as their impact on the critical attributes of the trastuzumab biosimilar. The insights gained from this study will serve as a foundation for the next stage formulation optimization, where selected formulations will be further assessed under expanded stress conditions to refine and validate their stability profiles.

## Material and methods

### Materials

Trastuzumab biosimilar in purified form was supplied by Catalent Biologics process development team (Bloomington, IN) at a concentration of ~ 20 mg/mL in pH 6.0, 10 mM histidine-HCl. Hydrochloric acid, sodium hydroxide, acetic acid, succinic acid, L-histidine, sodium phosphate monobasic, sodium chloride, L-arginine HCl, L-lysine HCl, sodium aspartate, L-glycine, sorbitol, sucrose, and β-mercaptoethanol were purchased from Sigma-Aldrich (St. Louis, MO) as ACS reagent grade. Polysorbate 20 and polysorbate 80 in the super refined grade are purchased from CRODA (Princeton, NJ). Iodoacetamide was obtained from ThermoFisher (Waltham, MA). Milli-Q water was used for all the experiments.

### Preparation of trastuzumab biosimilar in respective formulation conditions

Stock buffer solutions were prepared at 200 mM (i.e., 10X of their target formulation concentration) or 400 mM (20X) at the desired pH. They were checked for pH and osmolality and filtered through 0.22 μm filter prior to use. Buffers were labeled as Na-acetate, Na-succinate, histidine-HCl, and Na-phosphate in this study to simplify the discussion. Stock excipient modifier solutions were prepared at 5X of their target formulation concentration in water and filtered through 0.22 μm filter prior to use. Excipient modifiers were labeled as arginine-HCl, lysine-HCl, Na-aspartate, NaCl, glycine, sorbitol, sucrose to simplify the discussion. Control formulation without excipient modifier was labeled as no modifier. Final formulation buffer for buffer exchange was prepared by diluting corresponding stock buffer and stock excipient solutions in 96-well deep well plates. Preparations of buffer plates were performed with TECAN Freedom EVO100 (Switzerland). Target buffer concentration in the final solution is 20 mM. Target concentration of excipient modifiers such as arginine-HCl, lysine-HCl, Na-aspartate, NaCl is 150 mM in the final solution while target concentration for glycine, sorbitol, sucrose is 300 mM.

Buffer exchange and concentration of the trastuzumab biosimilar solution were performed in 24 or 96-well 30kD MWCO regenerated cellulose membrane filter plates using Big Tuna (UNchained Labs, Pleasanton, CA) automated buffer exchange system inside a customized enclosure to minimize particle contamination. A typical buffer exchange was performed with 60 psi chamber pressure at room temperature targeting 33% volume removal per cycle for up to five diafiltration volumes. Medical grade nitrogen supply was used to pressurize the chamber. High concentration sample preparation involved the following steps: 1) concentrating the trastuzumab biosimilar supply to ~ 75 mg/mL solution in a 24-well plate using water as a replacement solution when necessary, 2) combining the ~ 75 mg/mL solution from the 24-well plate and aliquoting to a 96-well plate, 3) performing buffer exchange into respective formulation matrixes using the 96-well plate, 4) concentrating to ~ 150 mg/mL solution or maximum concentration in the 96-well plate. The buffer exchange filtration flow rate at 75 mg/mL was reported as MidCon_FR while the maximum concentration reached in final concentration step was reported as Conc_Limit. Additional buffer exchange experiments at 20 mg/mL in 96-well plate were performed and the resulting buffer exchange filtration flow rate was reported as LowCon_FR. Samples were used directly after buffer exchange without additional composition adjustment to account for potential Donnan effect at high concentrations.

### Concentration control and stability setup

Protein concentration after buffer exchange and final concentration step was measured using Lunatic (Unchained Labs, Pleasanton, CA) HT UV/Vis reader. For concentrations below 40 mg/mL, Lunatic plates were used. All samples with concentrations above 40 mg/mL were measured with high Lunatic plates. 2 μL of protein sample were used for all measurements. Samples for stability study were prepared by diluting the ~ 75 mg/mL sample directly to 5 mg/mL with respective formulation matrixes and filtered using AcroPrepAdv 0.2 μm WWPTFE 96-well filter plate (Pall Corporation, Port Washington, NY). They were then stored in Nunc 96-well polypropylene storage plates (ThermoFisher, Waltham, MA) and sealed by Heat Sealing Foil (VITL, Ashland, VA) using HeatSealer (Eppendorf, Enfield, CT). A secondary packaging such as a vacuum sealed static shielding bag (ULINE, Pleasant Prairie, WI) was further used to minimize evaporation. Stability study was conducted by placing the plates in calibrated incubator at 40 °C without humidity control for two weeks. Samples were then tested by stability-indicating assays including SE-HPLC, reduced and non-reduced CGE.

### Biophysical parameter measurement

Melting temperatures of the trastuzumab biosimilar were determined using UNcle (UNchained Labs, Pleasanton, CA). The differential scanning fluorimetry was performed by heating samples loaded into Uni capillary cells (~9 uL of 5 mg/mL sample) from 25 °C to 95 °C at 0.25 °C/min and monitoring the intrinsic tryptophan fluorescence. In brief, fluorescence emission spectra were collected from 300 nm to 430 nm by exciting at 266 nm and analyzed by UNcleAnalysis to report barycentric mean (BCM) wavelength. Melting temperatures were determined from the inflection points of the BCM. For samples with multiple inflection points, the first inflection point (T_m1_) was reported as melting temperature in the study and likely corresponded to the unfolding of CH_2_ domain.

Protein–protein interaction was assessed by diffusion interaction parameter (k_D_) using an adapted protocol [23]. Briefly, a series of trastuzumab biosimilar solutions were prepared at 0 mg/mL, 1 mg/mL, 2 mg/mL, 3 mg/mL, 4 mg/mL, 6 mg/mL, 8 mg/mL, and 10 mg/mL protein concentration by diluting the trastuzumab biosimilar solution into their respective formulation buffers. Diffusion coefficients of the trastuzumab biosimilar in each respective formulation buffer were determined by dynamic light scattering using Wyatt DynaPro III (Wyatt, Santa Barbara, CA) at 25 °C. Diffusion interaction parameter (k_D_) was determined from the slope of the concentration response of normalized diffusion coefficients.

Apparent solubility of the trastuzumab biosimilar in formulations was assessed by an adapted PEG precipitation assay [22, 23]. Briefly, stock solution of PEG-6000 was prepared in water at 50% (w/v). They were then combined with 20 mg/mL trastuzumab biosimilar solution, 5X excipient modifier stocks, and 20X buffer stocks to create a series of trastuzumab biosimilar solution at 1 mg/mL of protein concentration with increasing percentage of PEG-6000 from 0% to 35% (w/v), with 5% increment. Samples were incubated at room temperature for two hours before analysis. Optical density at 350 nm (OD_350_) of the solution was analyzed by Agilent Cytation 5 microtiter plate reader (Agilent, Santa Clara CA) and was reported as solution turbidity. PEG-6000 concentration at the onset of the turbidly titration curve was reported as the relative solubility of the protein in the formulation buffer.

### Viscosity measurements

Samples for viscosity measurement were prepared by diluting the ~ 150 mg/mL trastuzumab biosimilar solution with respective formulation buffers to 125 mg/mL. The concentrations for viscosity measurement were tightly controlled at 126.6 ± 2.2 mg/mL for the 96 samples on the microtiter plate. 26 μL of each solution was then transferred into glass HPLC vials with integrated insert (Waters, Milford, MA) and stored in autosampler at 10 °C before analysis. Sample viscosities were measured on B05 chip at 20 °C using VROC Initium One Plus (Rheosense, San Ramon, CA). Standard protocols from the instrument to optimize pressure signal and test shear rate were used. Results were reported as the average of 10 measurements in the shear rate range 10^3^-10^4^ s^−1^. The system was cleaned using the vendor recommended aqueous solvent group including a series of washes by 1x PBS, 1% Aquet solution, and acetone.

### Analysis of size variants by size-exclusion ultra performance liquid chromatography (SE-UPLC)

Size variant analysis by SE-UPLC was performed using ACQUITY Premier Protein SEC Column (250 Å, 1.7 μm, 4.6 x 150 mm) (Waters, Milford MA) on ACQUITY H-Class UPLC with quaternary pump (Waters, Milford MA) and UV absorbance detection at 214 and 280 nm. Separation was performed at 25 °C under isocratic conditions at a flow rate of 0.3 mL/min with mobile phases containing 50 mM sodium phosphate (pH 7.0) and 150 mM sodium chloride. Before analysis, samples were diluted to 1 mg/mL with the mobile phase and centrifuged at 3220 x g for 10 min at 10 °C. Chromatography data were integrated and analyzed by Empower 3. High molecular weight (HMW), monomer, and low molecular weight (LMW) were reported as the percentage of total peak area. The net increase in the value after stability study was determined by subtracting the value of samples incubating 40 °C after 2 weeks from those of initial time points. These values determined from SE-UPLC were labeled in the study as D_HMW and D_LMW.

### Analysis of IgG purity by non-reduced and reduced capillary gel electrophoresis (NR/R-CGE)

Reduced and non-reduced SDS capillary gel electrophoresis experiments were performed on a Maurice instrument from ProteinSimple (San Jose, CA). Before analysis, the mAb samples were first diluted to 1 mg/mL with Milli-Q water and then diluted to final concentration of 0.25 mg/mL with the sample analysis buffer (Maurice CE-SDS PLUS 1X Sample Buffer, ProteinSimple). To 50 μL of the above samples, 2 μL of internal standard (Maurice CE-SDS 25x Internal Standard, ProteinSimple) was added. In addition, for non-reduced samples, 2.5 μL of 250 mM iodoacetamide was added and mixed by pipetting. For reduced samples, 2.5 μL of 14.2 M β-mercaptoethanol (Sigma Aldrich) was added and mixed by pipetting. The final mixture was heated at 70 °C for 10 minutes in ThermoMixer equipped with PCR plate adaptor (Eppendorf) and then immediately cooled. Cooled samples were mixed by pipetting, centrifuged at 2000 x g for 10 minutes, and stored in instrument autosampler before analysis. CE-SDS PLUS cartridges (ProteinSimple) used for analysis were prepared, conditioned, and washed following product protocols prior to use. Samples were loaded into cartridges by electrokinetic injection using 4600 V for 20 sec. Separation was performed using 5750 V for 35 min for non-reduced samples and for 25 min for reduced samples. Electropherogram data were integrated and analyzed by Compass for iCE (ProteinSimple). Fragmentation, intact IgG, and HMW were reported as the percentage of total peak area for NR-CGE. Fragmentation, light chain (LC), heavy chain (HC), and HMW were reported as the percentage of total peak area for R-CGE. The net increase in the value after stability study was determined by subtracting the value of samples incubating 40 °C after 2 weeks from those of initial time points. These values determined from CGE were labeled in the study as D_NR_HMW, D_NR_Frag, D_R_HMW, and D_R_Frag.

### Regression model and statistical analysis

Multiple linear regression and correlation analysis were performed using JMP 17 software package (SAS Institute Inc., Cary, NC). Pearson correlation coefficients were obtained by performing multivariate correlation analysis using row-wise estimation methods. Strength of correlation was grouped into strong correlation (0.7 ≤ |r| ≤ 1), moderate correlation (0.3 ≤ |r| < 0.7), and weak correlation (0 < |r| < 0.3).

For regression analysis, input variables included pH, buffer type, excipient modifier type, and their two-way cross interactions. A quadratic term of pH (pH x pH) was included as input variables for the fitting model of fragmentation (D_LMW). pH is a continuous factor while the types of buffer and excipient modifier are categorical factors. Output dependent variables for the models included biophysical parameters (T_m_, k_D_ etc.) as well as measurements from stability-indicating assays such as SE-UPLC and N/NR-CGE. The regression model was constructed for each individual output variable and required customized selection of input variables especially regarding cross interactions. This process involved first fitting a model with all individual factors and two-way interactions, then trimming the factors that were not significant based on the ANOVA tables after statistical tests of effects (i.e., removing effects with p > 0.01). In this way, the simplest models that can explain the most variability were used to interpretate the underlying trend in data. Standard least square analysis was used to perform the model fitting and obtain model parameters. The overall strength of the regression model was evaluated using R^2^ and graphically assessed through actual versus predicted results. To determine the contribution of each factor to the model, scaled parameter estimates were then used to rank model parameters and determine the factors with the most importance [11, 12]. Centering and scaling of continuous variables were done by the software to remove the variation in units and range of the factors. After the factor transformation, they were normalized to the range of 2, with a mean at 0. Partial t test was performed to determine the significance of each parameter in the model. Only factors that are statistically highly significant (e.g., p < 0.001) were included in the discussion. As the model is linear and continuous factors are normalized to the same range, higher absolute value of the scaled parameter estimates indicates higher importance in determining model response.

## Abbreviations

Monoclonal antibody (mAb); High-throughput (HT); Ultrafiltration-diafiltration (UF/DF); Critical quality attribute (CQA); Melting temperature (T_m_); Diffusion interaction parameter (k_D_); Differential scanning fluorimetry (DSF); Protein–protein interaction (PPI); Flow rate (FR); High molecular weight (HMW); Low molecular weight (LMW); Size-exclusion ultra performance liquid chromatography (SE-UPLC); Non-reduced capillary gel electrophoresis (NR-CGE); Reduced capillary gel electrophoresis (R-CGE); Intravenous (IV); Subcutaneous (SC)

## Supplementary Material

mAb_excipient_compatiblity_manuscript_supplements_accepted_revision_cleaned_tbae028

## Data Availability

The data underlying this article are available in the article and in its online supplementary material.
