# Supplementary material for: Leveraging high-throughput analytics and automation to rapidly develop high-concentration mAb formulations: integrated excipient compatibility and viscosity screening
Source: Antib Ther. 2024 Oct 12;7(4):335–50. doi: 10.1093/abt/tbae028 (PMC11646310; doi:10.1093/abt/tbae028)

SUPPLEMENTAL MATERIALS

**Leveraging High-Throughput Analytics and Automation to Rapidly Develop High-Concentration mAb Formulations: Integrated Excipient Compatibility and Viscosity Screening**

Lun Xin^1^, Lan Lan^1^, Mourad Mellal^2^, Nathan McChesney^1^, Robert Vaughan^1^, Claudia Berdugo^1^, Yunsong Li^1^, Jingtao Zhang^2^*

Affiliations : ^1^Product Development, Catalent Pharma Solutions, Bloomington, IN, USA; ^2^Product Development, Catalent Pharma Solutions, Somerset, NJ, USA

*Corresponding author: Jingtzhang@gmail.com

**Table S1**: Layout of the 96 well plate for the integrated excipient compatibility and viscosity screening of trastuzumab biosimilar formulations. The composition of a formulation in the 96 well plate can be determined by orthogonally pairing the components and levels shown in the table headers. For example, each formulation will consist of a 20 mM buffer at the target pH and an excipient modifier at the described concentration in the table. Control formulations with only buffer and no excipient modifier are labeled as No Modifier. Viscosity values (unit in cP) of 125 mg/mL trastuzumab biosimilar in these formulation conditions were shown in the table as well. A heatmap with green-yellow-orange-red color scale was used to present the viscosity data with lowest values represented by green and the highest values represented by red for their desirability.

| Buffer (pH)  /Excipient Modifiers | 20 mM Na-Acetate | | | 20 mM Na-Succinate | | | 20 mM Histidine-HCl | | | 20 mM Na-Phosphate | | |
| --- | --- | --- | --- | --- | --- | --- | --- | --- | --- | --- | --- | --- |
|  | 4.5 | 5.0 | 5.5 | 4.5 | 5.0 | 5.5 | 5.5 | 6.0 | 6.5 | 6.5 | 7.0 | 7.5 |
| 150 mM Arginine-HCl | 5.0 | 5.2 | 4.9 | 5.3 | 5.2 | 5.1 | 5.6 | 5.3 | 5.4 | 5.6 | 5.8 | 6.4 |
| 150 mM Lysine-HCl | 5.3 | 5.3 | 5.5 | 5.3 | 5.3 | 5.7 | 5.6 | 5.9 | 6.0 | 6.3 | 6.4 | 6.9 |
| 150 mM Na-Aspartate | 4.9 | 4.6 | 5.1 | 4.9 | 5.7 | 5.6 | 5.3 | 5.5 | 6.1 | 6.4 | 6.7 | 7.8 |
| 150 mM NaCl | 5.4 | 5.2 | 5.3 | 5.8 | 5.6 | 6.5 | 5.8 | 5.4 | 5.7 | 6.4 | 7.1 | 7.7 |
| 150 mM Glycine | 4.4 | 4.4 | 5.0 | 4.8 | 4.8 | 5.3 | 4.8 | 5.2 | 6.1 | 6.4 | 7.4 | 7.8 |
| 300 mM Sorbitol | 5.4 | 5.4 | 6.0 | 5.4 | 5.5 | 6.3 | 5.9 | 6.2 | 8.5 | 8.4 | 7.7 | 8.7 |
| 300 mM Sucrose | 7.1 | 7.4 | 7.7 | 7.3 | 7.1 | 8.7 | 8.0 | 8.8 | 10.9 | 10.7 | 13.6 | 13.1 |
| No Modifier | 4.4 | 4.5 | 4.8 | 4.6 | 4.8 | 6.0 | 5.1 | 6.2 | 6.8 | 7.6 | 8.6 | 9.7 |

**Table S2**: A comparison of the resource required and number of screened samples by two different approaches (high throughput screening vs. sequential screening) for high concentration mAb formulation development. The hypothetical sequential screening includes a pH/buffer screening, an excipient screening, and a high concentration screening (Figure 1A), conducted manually in 2R vials. Numbers for the sequential screening are estimates based on historical experiences and vary based on study design.

|  | HT screening in the manuscript | Hypothetical sequential screening of similar scopes |
| --- | --- | --- |
| Total screened samples | 96 | 30-60 |
| Material requirement (g) | 2 | 8-20 |
| Time needed (weeks) | 6 | 12-16 |


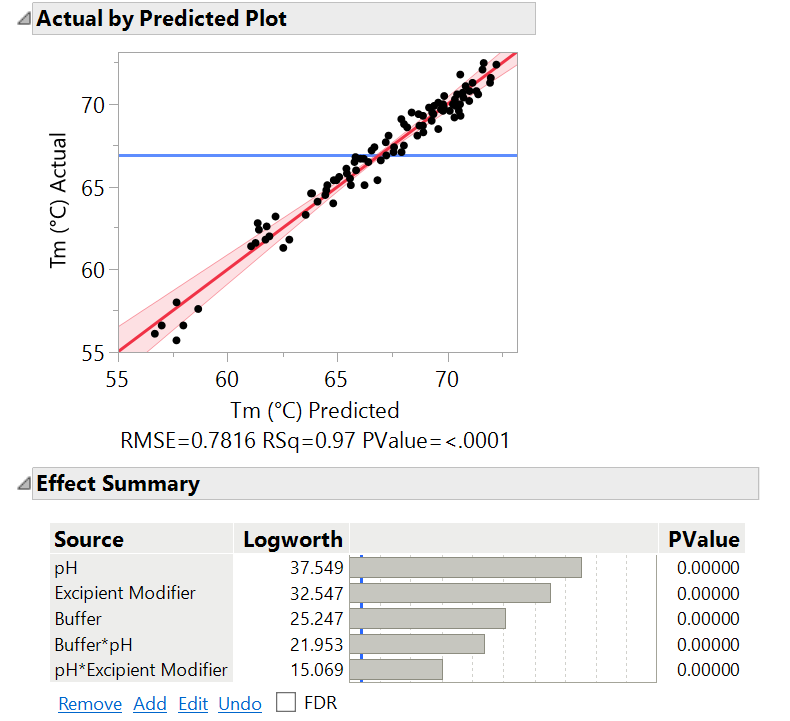


**Figure S1**: Actual by predicted plot for the multiple linear regression model of the trastuzumab biosimilar’s melting temperature (T_m_) in 96 formulation conditions. Regression model was constructed using individual factors (pH, buffer, and excipient modifier) as well as selected two-way interactions (pH x buffer, pH x excipient modifier). The lack of overlap between the red shaded area (95% confidence region) with the blue line (null hypothesis that the response is independent of the factors) shows that the whole model is highly significant. P value of the whole model determined from F test, R^2^, and root mean square error (RMSE) was also shown. All effects in the model are very significant (p < 0.01).

**Table S3**: Scaled parameter estimates for the multiple linear regression model of the trastuzumab biosimilar’s melting temperature (T_m_) in 96 formulation conditions. Regression model was constructed using individual factors (pH, buffer, and excipient modifier) as well as selected two-way interactions (pH x buffer, pH x excipient modifier). To remove the variation in units and range of the factors, continuous factors were normalized to the same range of 2, with a mean at 0. For example, pH was centered and scaled by subtracting their mean (5.75) from the pre-conversion value and dividing the result by range/2 (1.5). Partial t test was performed to determine the significance of each individual parameter in the model, with t ratio and p value shown. Only factors that are statistically highly significant (e.g., p < 0.001) were included in the discussion. Scaled parameter estimates in the table are sorted based on their value. As the model is linear and factors are normalized to the same range, the higher absolute value of the scaled parameter estimates indicates higher importance in determining model response.


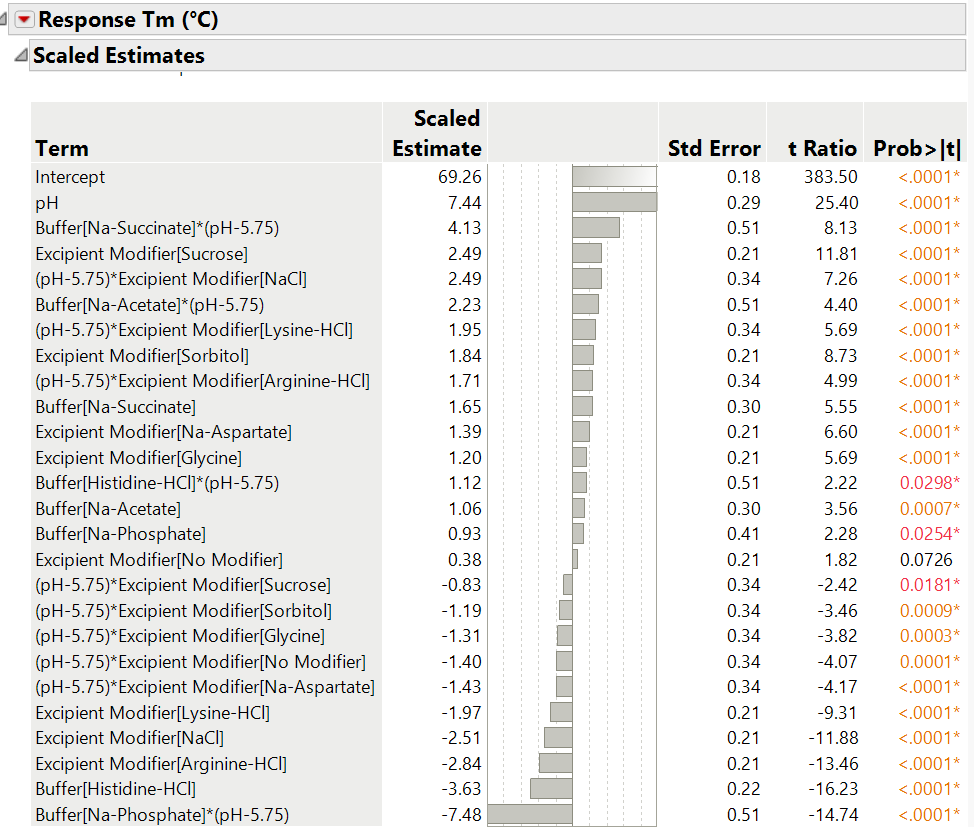


| A  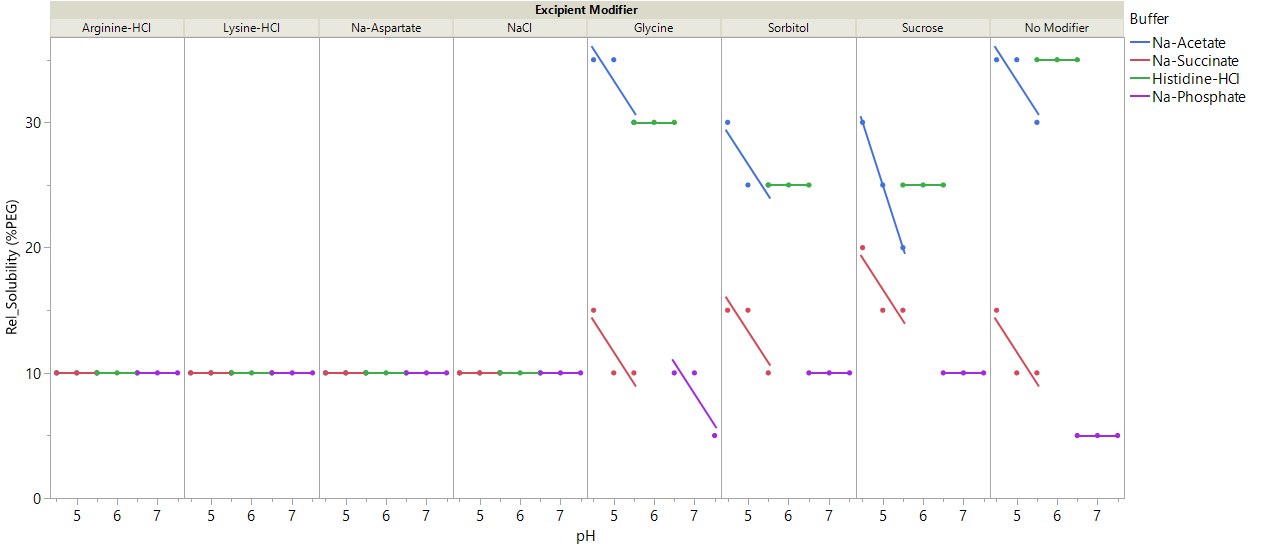 |
| --- |
| B  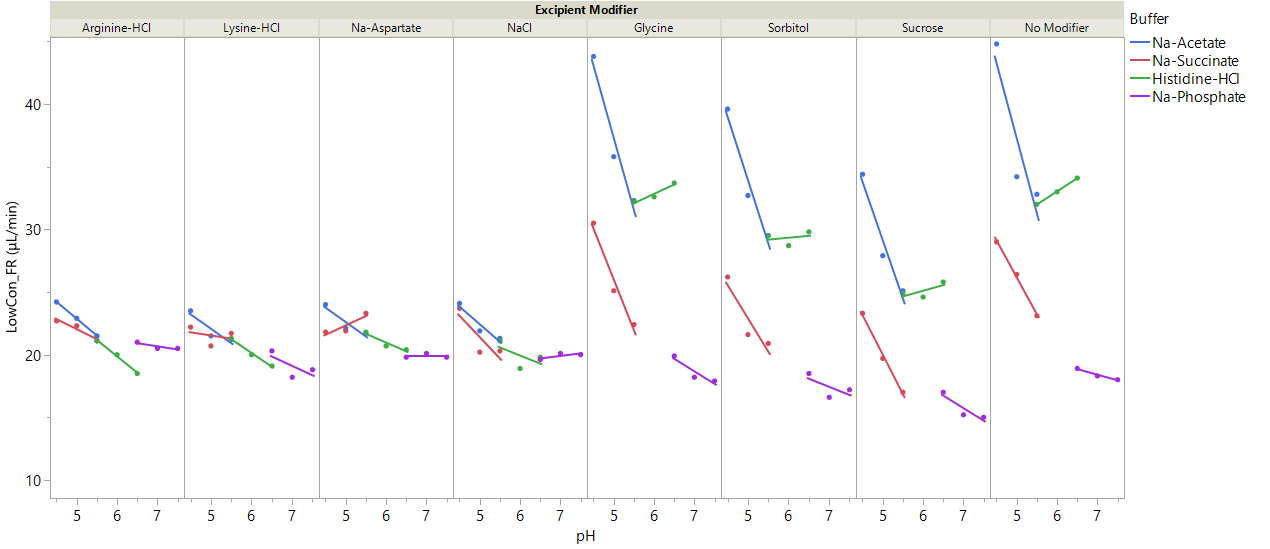 |
| C  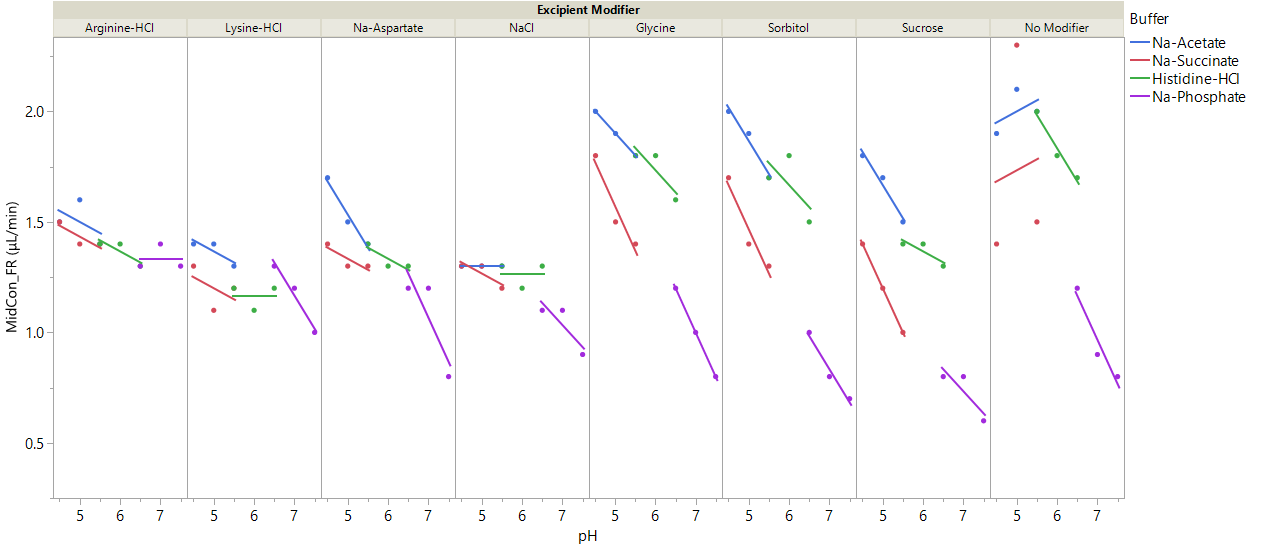 |
| D  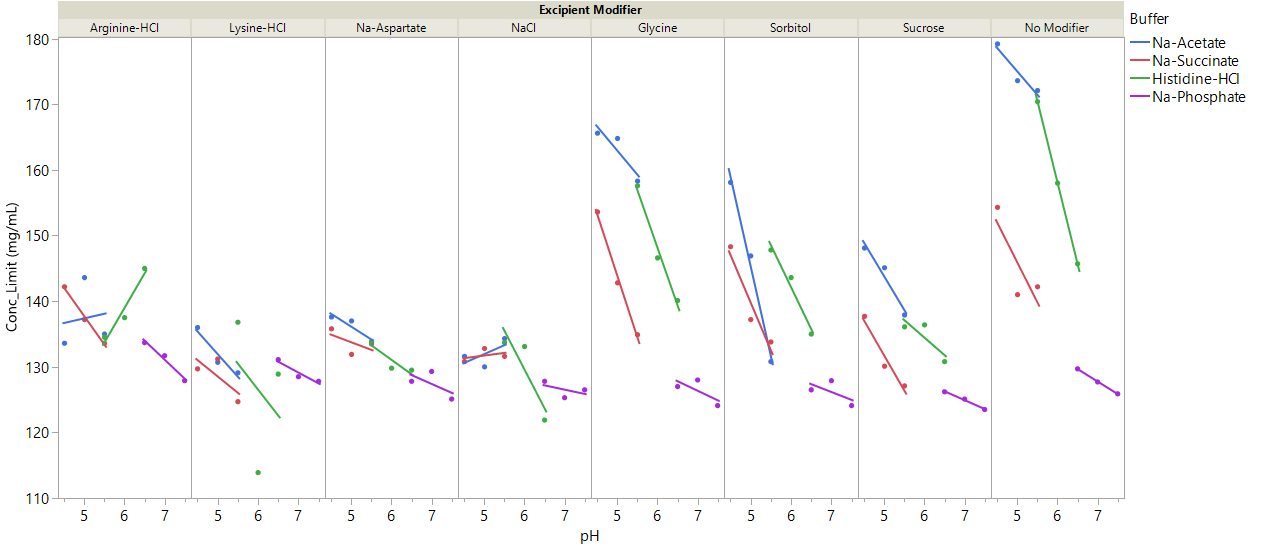 |
|  |
| **Figure S2**: Impact of formulation conditions on relative solubility and buffer exchange in process measurements of the trastuzumab biosimilar in different formulation conditions (Panel A: relative solubility as measured by PEG-6000 precipitation; Panel B: buffer exchange filtration flow rate at 20 mg/mL; Panel C: buffer exchange filtration flow rate at 75 mg/mL; Panel D: maximum concentration reached in final concentration step). Data are shown as experimental values (solid symbols) overlaid with fitted lines to guide data reading. Each formulation consists of a 20 mM buffer at the target pH and an excipient modifier (see methods for details on formulation conditions). Control formulation with only buffer and no excipient modifier is labeled as No Modifier. Formulations consisting of the same buffer are labeled using the same color (blue: Na-Acetate; red: Na-Succinate; green: Histidine-HCl; purple: Na-Phosphate). |


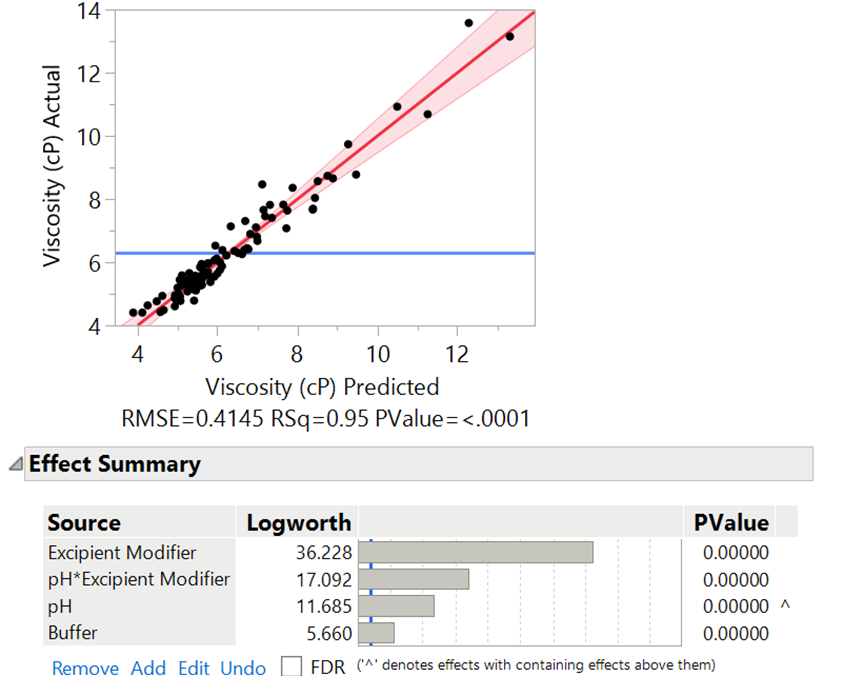


**Figure S3**: Actual by predicted plot for the multiple linear regression model of the trastuzumab biosimilar’s viscosity in 96 formulation conditions. Regression model was constructed using individual factors (pH, buffer, and excipient modifier) as well as a two-way interaction (pH x excipient modifier). The lack of overlap between the red shaded area (95% confidence region) with the blue line (null hypothesis that the response is independent of the factors) shows that the whole model is highly significant. P value of the whole model determined from F test, R^2^, and root mean square error (RMSE) was also shown. All effects in the model are very significant (p < 0.01).

**Table S4**: Scaled parameter estimates for the multiple linear regression model of the trastuzumab biosimilar’s viscosity in 96 formulation conditions. Regression model was constructed using individual factors (pH, buffer, and excipient modifier) as well as a two-way interaction (pH x excipient modifier). To remove the variation in units and range of the factors, continuous factors were all normalized to the same range of 2, with a mean at 0. For example, pH was centered and scaled by subtracting their mean (5.75) from the pre-conversion value and dividing the result by range/2 (1.5). Partial t test was performed to determine the significance of each individual parameter in the model, with t ratio and p value shown. Only factors that are statistically highly significant (e.g., p < 0.001) were included in the discussion. Scaled parameter estimates in the table are sorted based on their value. As the model is linear and factors are normalized to the same range, the higher absolute value of the scaled parameter estimates indicates higher importance in determining model response.


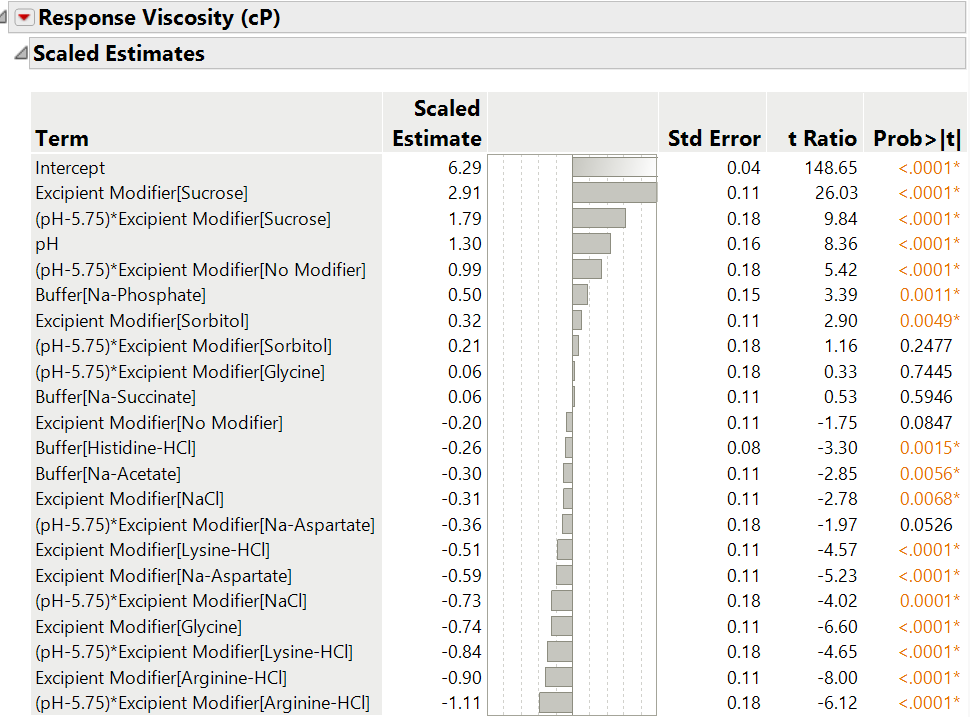


| A  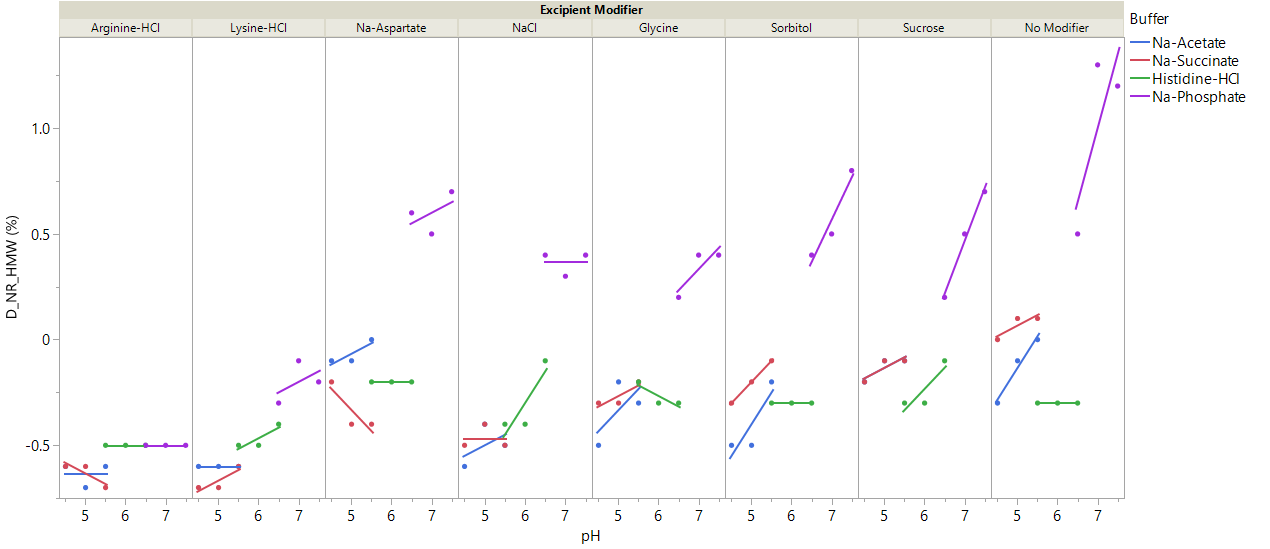 |
| --- |
| B  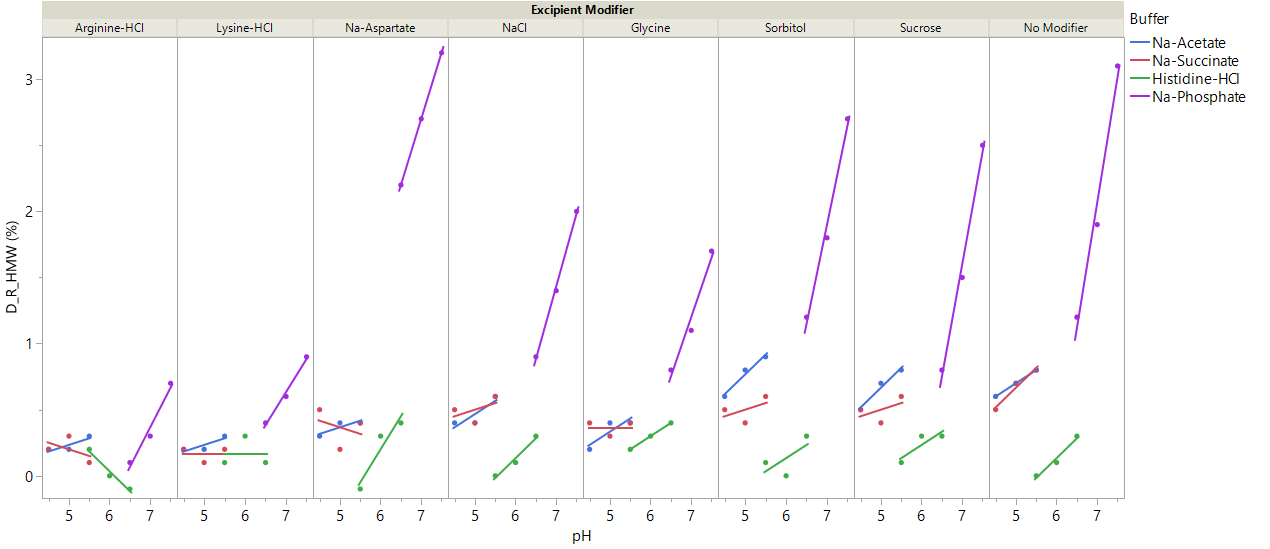 |
|  |
| **Figure S4**: Impact of formulation conditions on the HMW aggregation of the trastuzumab biosimilar. Trastuzumab formulations at 5 mg/mL were stressed at 40 °C for 2 weeks and subsequently subjected to NR/R-CGE to determine the net increase in HMW value (Panel A: net increase in HMW as determined by NR-CGE (D_NR-HMW); Panel B: net increase in HMW as determined by R-CGE (D_R_HMW). Data are shown as experimental values (solid symbols) overlaid with fitted lines to guide data reading. Each formulation consists of a 20 mM buffer at the target pH and an excipient modifier (see methods for details on formulation conditions). Control formulation with only buffer and no excipient modifier is labeled as No Modifier. Formulations consisting of the same buffer are labeled using the same color (blue: Na-Acetate; red: Na-Succinate; green: Histidine-HCl; purple: Na-Phosphate). |

| 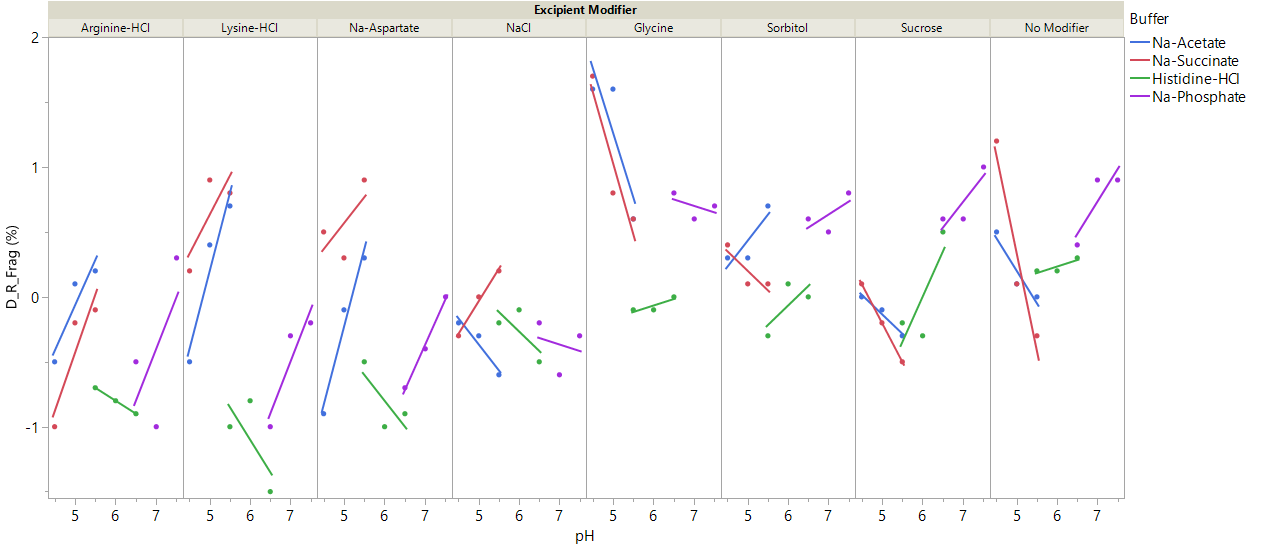 |
| --- |
|  |
| **Figure S5**: Impact of formulation conditions on the fragmentation of the trastuzumab biosimilar. Trastuzumab formulations at 5 mg/mL were stressed at 40 °C for 2 weeks and subsequently subjected to R-CGE to determine the net increase in fragmentation value (D_R_Frag). Data are shown as experimental values (solid symbols) overlaid with fitted lines to guide data reading. Each formulation consists of a 20 mM buffer at the target pH and an excipient modifier (see methods for details on formulation conditions). Control formulation with only buffer and no excipient modifier is labeled as No Modifier. Formulations consisting of the same buffer are labeled using the same color (blue: Na-Acetate; red: Na-Succinate; green: Histidine-HCl; purple: Na-Phosphate). |


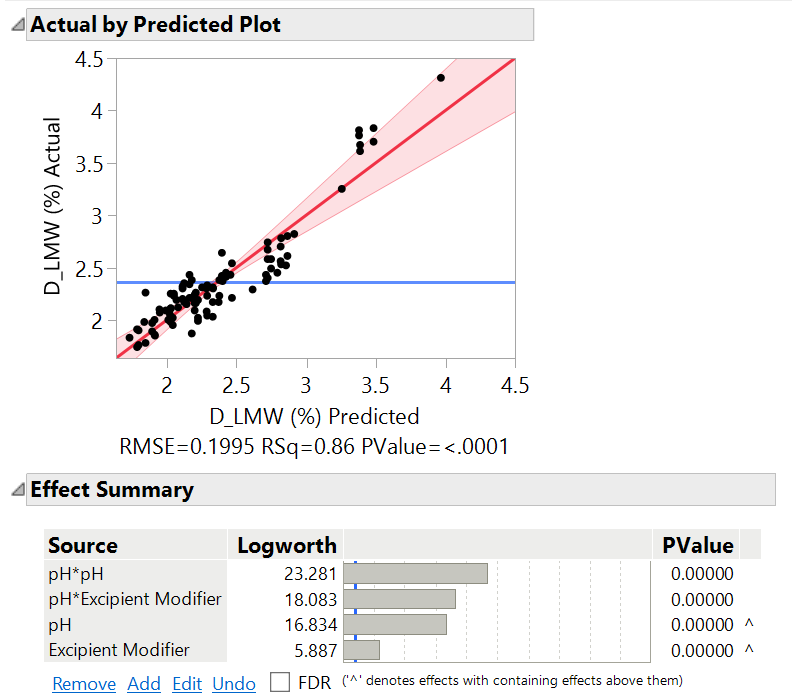


**Figure S6**: Actual by predicted plot for the multiple linear regression model of the trastuzumab biosimilar’s D_LMW in 96 formulation conditions. Regression model was constructed using individual factors (pH and excipient modifier), a two-way interaction (pH x excipient modifier), and a quadratic term of pH (pH x pH). The lack of overlap between the red shaded area (95% confidence region) with the blue line (null hypothesis that the response is independent of the factors) shows that the whole model is highly significant. P value of the whole model determined from F test, R^2^, and root mean square error (RMSE) was also shown. All effects in the model are very significant (p < 0.01).

**Table S5**: Scaled parameter estimates for the multiple linear regression model of the trastuzumab biosimilar’s D_LMW in 96 formulation conditions. Regression model was constructed using individual factors (pH and excipient modifier), a two-way interaction (pH x excipient modifier), and a quadratic term of pH (pH x pH). To remove the variation in units and range of the factors, continuous factors were all normalized to the same range of 2, with a mean at 0. For example, pH was centered and scaled by subtracting their mean (5.75) from the pre-conversion value and dividing the result by range/2 (1.5). Partial t test was performed to determine the significance of each individual parameter in the model, with t ratio and p value shown. Only factors that are statistically highly significant (e.g., p < 0.001) were included in the discussion. Scaled parameter estimates in the table are sorted based on their value. As the model is linear and factors are normalized to the same range, the higher absolute value of the scaled parameter estimates indicates higher importance in determining model response.


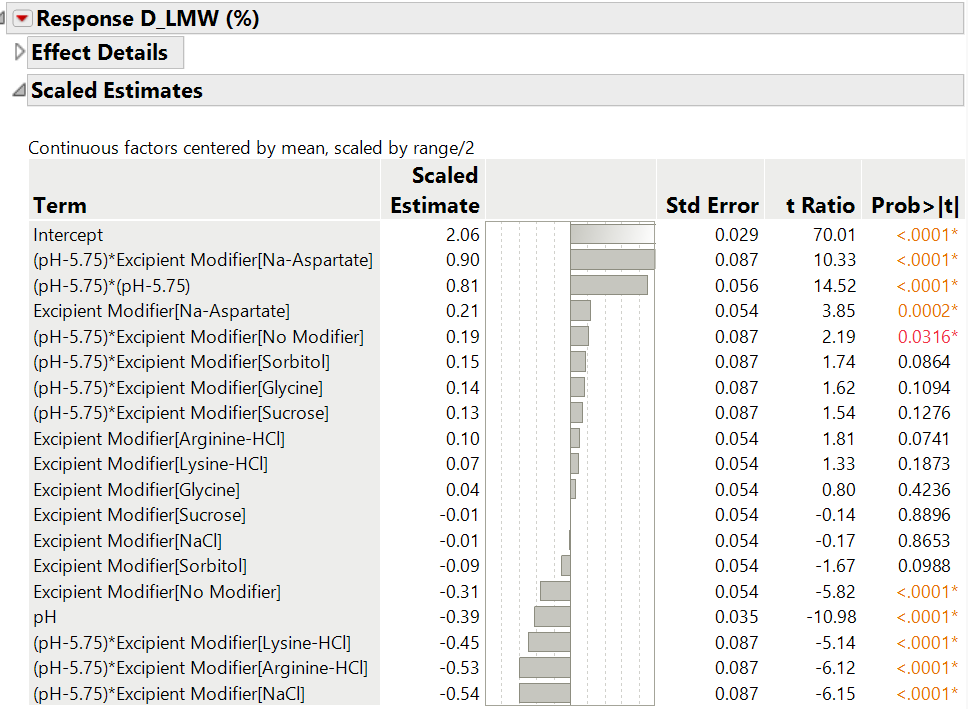

Supplement: mAb_excipient_compatiblity_manuscript_supplements_accepted_revision_cleaned_tbae028 [file mab_excipient_compatiblity_manuscript_supplements_accepted_revision_cleaned_tbae028.docx]
